# Development of a multidisciplinary service-delivery framework for venous thromboembolism prevention in hospitalized medical patients with lung cancer: a mixed-methods modified Delphi study

**DOI:** 10.3389/fcvm.2026.1935436

**Published:** 2026-10-28

**Authors:** Yanhong Meng, Sijia Zhong, Yuhang Ding, Pengnan Xu, Yongsheng Wang, Jinping Zhang, Qiuhui Wu

**Affiliations:** 1 Department of Pharmacy, Nanjing Drum Tower Hospital, School of Basic Medicine and Clinical Pharmacy, China Pharmaceutical University, Nanjing, China; 2 Guangxi Hospital Division of the First Affiliated Hospital, Sun Yat-sen University, Nanning, China; 3 Department of Pharmacy, Nanjing Drum Tower Hospital, Affiliated Hospital of Medical School, Nanjing University, Nanjing, China; 4 China Hospital Reform and Development Research Institute of Nanjing University, Nanjing Drum Tower Hospital, Nanjing, China

**Keywords:** Delphi technique, health services research, implementation science, lung cancer, patient safety, quality indicators, venous thromboembolism

## Abstract

**Background:**

Venous thromboembolism (VTE) is a common and potentially preventable complication in patients with lung cancer. Although clinical guidelines recommend risk assessment and appropriate prophylaxis, hospitals often lack operational frameworks for translating these recommendations into multidisciplinary workflows, quality monitoring, and continuous improvement. This study aimed to develop a hospital-based multidisciplinary service-delivery framework for VTE prevention and management among hospitalized medical patients with lung cancer.

**Methods:**

A mixed-methods modified Delphi study was conducted. Candidate framework items were generated from a structured literature review and semi-structured interviews with physicians, nurses, clinical pharmacists, and hospital managers, and were organized using Donabedian’s Structure–Process–Outcome model. A two-round Delphi survey was then conducted with 15 experts from respiratory medicine, oncology, clinical pharmacy, nursing management, and hospital quality management. Experts rated the importance and feasibility of each candidate item, and the framework was refined using predefined consensus criteria and qualitative feedback.

**Results:**

The response rate was 100% in both Delphi rounds. Expert authority coefficients were 0.943 and 0.953 in rounds 1 and 2, respectively. Kendall's W ranged from 0.249 to 0.292, indicating low-to-moderate concordance despite statistically significant tests. The final framework included 3 first-level domains, 11 second-level components, and 34 third-level indicators covering structural capacity, care processes, and outcome monitoring. Importance ratings were generally higher than feasibility ratings, particularly for indicators requiring information system support, multidisciplinary coordination, and post-discharge continuity of care.

**Conclusion:**

This study developed a preliminary, consensus-based multidisciplinary service-delivery framework for hospital-based VTE prevention in patients with lung cancer. The framework may support the operationalization of guideline recommendations, standardization of care processes, and prioritization of quality improvement efforts. Prospective implementation studies are needed to evaluate its feasibility, transferability, and impact on care quality and patient outcomes.

## Introduction

Lung cancer remains a leading cause of cancer-related morbidity and mortality worldwide, and venous thromboembolism (VTE) is one of the most important preventable complications in this population. Patients with lung cancer are at increased risk of VTE because of tumor-related hypercoagulability, anticancer treatment, hospitalization, reduced mobility, invasive procedures, and comorbid conditions. VTE can prolong hospital stay, increase healthcare costs, interrupt anticancer treatment, and worsen prognosis (1, 2). From a public health and patient safety perspective, effective VTE prevention in patients with lung cancer requires not only appropriate clinical decisions but also reliable systems for risk assessment, prophylaxis delivery, patient education, follow-up, and quality monitoring.

Several national and international guidelines recommend routine VTE risk assessment and appropriate prophylaxis for patients with cancer (3–5). However, guideline availability does not necessarily lead to consistent implementation in routine hospital care. In practice, gaps may occur at multiple levels, including incomplete risk assessment, uncertainty about professional responsibilities, insufficient patient education, limited medication safety monitoring, weak post-discharge continuity, and inadequate audit-feedback mechanisms. These gaps suggest that the challenge is not only clinical but also organizational and implementation-related (6).

For hospitalized patients with lung cancer, VTE prevention is a multidisciplinary service-delivery problem. It requires coordination among respiratory physicians, oncologists, nurses, clinical pharmacists, hospital managers, and quality management teams. It also requires institutional protocols, electronic risk assessment tools, standardized workflows, medication safety processes, patient-facing educational materials, and measurable quality indicators. Donabedian's Structure–Process–Outcome model provides a useful conceptual framework for organizing these components by linking institutional capacity, care processes, and measurable outcomes (7).

Delphi methods are widely used to develop consensus-based frameworks and quality indicators when evidence is fragmented, practice is heterogeneous, and multidisciplinary judgment is required. However, Delphi-based framework development is strengthened when expert consensus is informed by empirical evidence and stakeholder perspectives rather than expert opinion alone (8). Therefore, this study combined a structured literature review, semi-structured interviews, and a two-round Delphi process to develop a preliminary, consensus-based multidisciplinary service-delivery framework for VTE prevention and management in hospitalized medical patients with lung cancer. The purpose was not to propose a new anticoagulant regimen, but to translate existing guideline recommendations into operational indicators that can support hospital-based quality improvement and future implementation research.

## Materials and methods

### Study design

This mixed-methods modified Delphi study developed a preliminary, consensus-based multidisciplinary service-delivery framework for venous thromboembolism (VTE) prevention and management in hospitalized medical patients with lung cancer. Donabedian's Structure–Process–Outcome (SPO) model informed the organization of candidate framework components addressing organizational structures, care processes, and quality monitoring (7). Framework development involved three stages: (1) generation of candidate items from a structured literature review and semi-structured interviews; (2) refinement through a two-round Delphi survey; and (3) statistical assessment of expert engagement, authority, and consensus. The reporting of the Delphi process was informed by the CREDES recommendations for conducting and reporting Delphi studies (8).

### Intended population and scope

The framework is intended for hospitalized medical patients with lung cancer, particularly those admitted to respiratory medicine or medical oncology services. Surgical admissions, brief admissions solely for chemotherapy infusion or minor procedures, ICU care, and end-of-life care require separate adaptation and are outside its current intended scope. Patients receiving therapeutic-dose anticoagulation are excluded from the pharmacological primary-prophylaxis denominator but may remain relevant to other framework components, such as medication safety and patient education.

The framework focuses on VTE prevention. Components addressing suspected deep vein thrombosis (DVT) or pulmonary embolism (PE) support early recognition, diagnostic referral, and escalation of care; they do not constitute a treatment pathway for established VTE.

### Research team

The study was conducted by a seven-member interdisciplinary team consisting of one associate chief physician, one chief pharmacist, one associate chief pharmacist, two senior nurses, and two graduate students. Except for the graduate students, all team members had more than 10 years of relevant clinical or research experience. The team was responsible for the literature review, interview design, development of the Delphi questionnaires, data collection, statistical analysis, and refinement of the final framework.

## Literature review

A structured literature search was conducted in PubMed, Web of Science, Ovid, CNKI, Wanfang, SinoMed, and VIP, from database inception to May 31, 2025. Search terms combined Medical Subject Headings and free-text terms related to lung cancer, venous thromboembolism, thromboprophylaxis, risk assessment, quality indicators, the Structure–Process–Outcome model, and the Delphi technique. The review was undertaken for item generation rather than effect estimation. Literature was eligible if it addressed VTE prevention in patients with lung cancer, reported prevention strategies, multidisciplinary workflows, quality indicators, or clinical practice guidelines/expert consensus statements relevant to cancer-associated thrombosis, and was published in Chinese or English. Two researchers independently screened the literature and extracted information on risk assessment tools, prophylaxis processes, multidisciplinary roles, patient education, follow-up requirements, medication safety, and quality monitoring. Disagreements were resolved through discussion with a senior investigator. Findings were used to identify preliminary framework domains and candidate indicators.

### Semi-structured interviews

To complement the literature review with practice-based perspectives, one-to-one semi-structured interviews were conducted between February and April 2025 at Nanjing Drum Tower Hospital using stratified purposive sampling. Eligible participants were physicians, nurses, clinical pharmacists, and hospital administrators directly involved in VTE prevention or management for patients with lung cancer. A total of 16 participants were interviewed, including 9 physicians, 3 nurses, 2 clinical pharmacists, and 2 hospital administrators (Table 1). The interview guide focused on professional roles, institutional resources, existing workflows, quality monitoring, barriers to implementation, multidisciplinary communication, patient education, medication safety, and priorities for service improvement.

**Table 1:** Characteristics of the interview participants (*N* = 16).

Characteristic	Category	*n*	Percentage (%)
Gender	Male	5	31.3
	Female	11	68.8
Age (years)	29–34	2	12.5
	35–44	10	62.5
	≥45	4	25.0
Work experience (years)	≤5	2	12.5
	6–14	8	50.0
	≥15	6	37.5
Educational background	Bachelor's degree	3	18.8
	Master's degree	7	43.8
	Doctoral degree	6	37.5
Professional title	Junior title	2	12.5
	Intermediate title	3	18.8
	Associate senior title	7	43.8
	Senior title	4	25.0
Profession	Physicians	9	56.3
	Nurses	3	18.8
	Pharmacists	2	12.5
	Administrators	2	12.5

Percentages are rounded to one decimal place and may not sum to 100%.

Interviews were conducted by graduate researchers with training in qualitative research methods who had no prior professional relationships with the participants. Written informed consent was obtained before each interview. Interviews lasted approximately 30–45 min and were audio-recorded, with accompanying field notes. Interview recordings were transcribed verbatim within 24 h and analyzed using NVivo 12.0, with participant recruitment continuing until analysis of successive interviews identified no substantially new categories relevant to the study objectives.

The Consolidated Framework for Implementation Research (CFIR) provided the initial coding framework (9). Two researchers independently pilot-coded three transcripts and compared their coding to develop a shared coding manual. They then independently coded the remaining transcripts. Disagreements were resolved through discussion, with unresolved differences referred to a third senior researcher. Findings were organized by CFIR domain and examined for barriers to and facilitators of VTE prevention. The research team subsequently considered these findings alongside the literature review to inform the content and wording of candidate framework items.

### Development of the Delphi questionnaire

The initial Delphi questionnaire was developed by integrating findings from the literature review, interview analysis, and the SPO framework. It included three sections: an introduction to the study purpose, an indicator rating section, and an expert profile section. In round 1, the questionnaire included 3 first-level domains, 11 second-level components, and 38 third-level candidate indicators. Experts rated each indicator on importance and feasibility using a 5-point Likert scale, where 1 indicated the lowest level and 5 indicated the highest. Free-text fields were provided after each indicator to collect suggestions for modification, deletion, merging, or addition. The round 2 questionnaire was revised based on quantitative ratings and qualitative comments from round 1 and contained 3 first-level domains, 11 second-level components, and 34 third-level indicators.

### Selection of experts

Experts were invited from respiratory medicine, medical oncology, nursing, clinical pharmacy, and hospital quality or administrative management. Eligibility criteria were as follows: relevant expertise and practical experience in VTE prevention or management for patients with lung cancer; an intermediate or higher professional title; a bachelor's degree or above; at least 10 years of work experience; and willingness to participate in both rounds of consultation. Fifteen experts were recruited from Nanjing Drum Tower Hospital, a tertiary hospital in Jiangsu Province, China, for the Delphi survey. Some had previously participated in the qualitative interviews.

### Modified Delphi procedures

Two rounds of Delphi consultation were conducted between April and June 2025. Questionnaires were distributed through WeChat, the Wenjuanxing platform, and face-to-face communication. Individual ratings were not disclosed to other panel members, but participants were identifiable to the research team through information collected for study administration. After round 1, the research team reviewed the quantitative ratings and written comments and revised the questionnaire. Before round 2, panelists received the revised questionnaire but were not provided with structured feedback on group ratings, summaries of panelists' comments, or their own previous scores. After each round, responses were collated and analyzed by the research team. Indicators were retained, revised, merged, or deleted according to predefined selection criteria and expert comments. Indicators that did not meet all quantitative thresholds were reviewed in light of their clinical relevance, service-delivery importance, and qualitative feedback. Such indicators were retained only when experts considered them important implementation priorities despite lower feasibility. Following the first round, one item was deleted, six items were merged into 3 items, and 12 items were revised. After the second round, no further modifications were made. The final framework consisted of 3 first-level domains, 11 second-level components, and 34 third-level indicators.

### Statistical analysis

Data were analyzed using Excel 2019 and SPSS version 26.0. Expert engagement was evaluated using the response rate of valid questionnaires. Expert authority was assessed using the authority coefficient (Cr), calculated as the mean of the judgment basis coefficient (Ca) and familiarity coefficient (Cs): Cr = (Ca + Cs)/2. The scoring criteria for Ca and Cs are shown in Tables 2, 3. Indicator ratings were summarized using means, standard deviations, and coefficients of variation (CVs) for both importance and feasibility. The predefined screening criteria were a mean importance score ≥4.00, a mean feasibility score ≥4.00, and a CV < 0.25 (10). Kendall's coefficient of concordance (W) was used to summarize overall agreement among experts. Interpretation focused on the magnitude of W, with *P* values reported as Supplementary Information. Statistical significance was not interpreted as evidence of strong consensus or framework validity.

**Table 2:** Scoring criteria for the judgment basis coefficient (Ca).

Judgment basis	Degree of influence		
	High	Moderate	Low
Practical experience	0.5	0.4	0.3
Theoretical analysis	0.3	0.2	0.1
Reference to domestic and international literature	0.1	0.1	0.1
Intuition	0.1	0.1	0.1

**Table 3:** Scoring criteria for the familiarity coefficient (Cs).

Degree of familiarity	Very familiar	Relatively familiar	Moderately familiar	Not very familiar	Unfamiliar
Assigned value	1	0.8	0.6	0.4	0.2

### Ethics approval

This study, including the semi-structured interviews and modified Delphi consultation, was approved by the Ethics Committee of Nanjing Drum Tower Hospital (approval number: 2025-0779-02) and was conducted in accordance with the Declaration of Helsinki. Before participation, all interviewees and experts were informed of the study objectives, content, and procedures. Participation was voluntary, and informed consent was obtained before data collection.

## Results

### Evidence review findings

Literature was identified and screened through systematic searches of Chinese- and English-language databases, as illustrated in Figure 1. Searches yielded 668 records from English-language databases (PubMed, Web of Science, and Ovid) and 1,287 records from Chinese-language databases (CNKI, Wanfang, SinoMed, and VIP), giving a total of 1,955 records. After removing 233 duplicates, 1,722 records remained for initial screening. Screening of titles, abstracts, and keywords excluded records that were unrelated to the topic (*n* = 1,202), involved an ineligible study population (*n* = 274), or were published in languages other than Chinese or English (*n* = 4), leaving 242 records for full-text assessment. A further 231 records were excluded because their content did not meet the eligibility criteria (*n* = 216), the full text was unavailable (*n* = 5), and they were judged by the investigators to be insufficiently suitable or rigorous for candidate-item generation (*n* = 10). Ultimately, 11 publications, including guidelines, were included. Their characteristics were summarized in Table 4.

**Figure 1:**
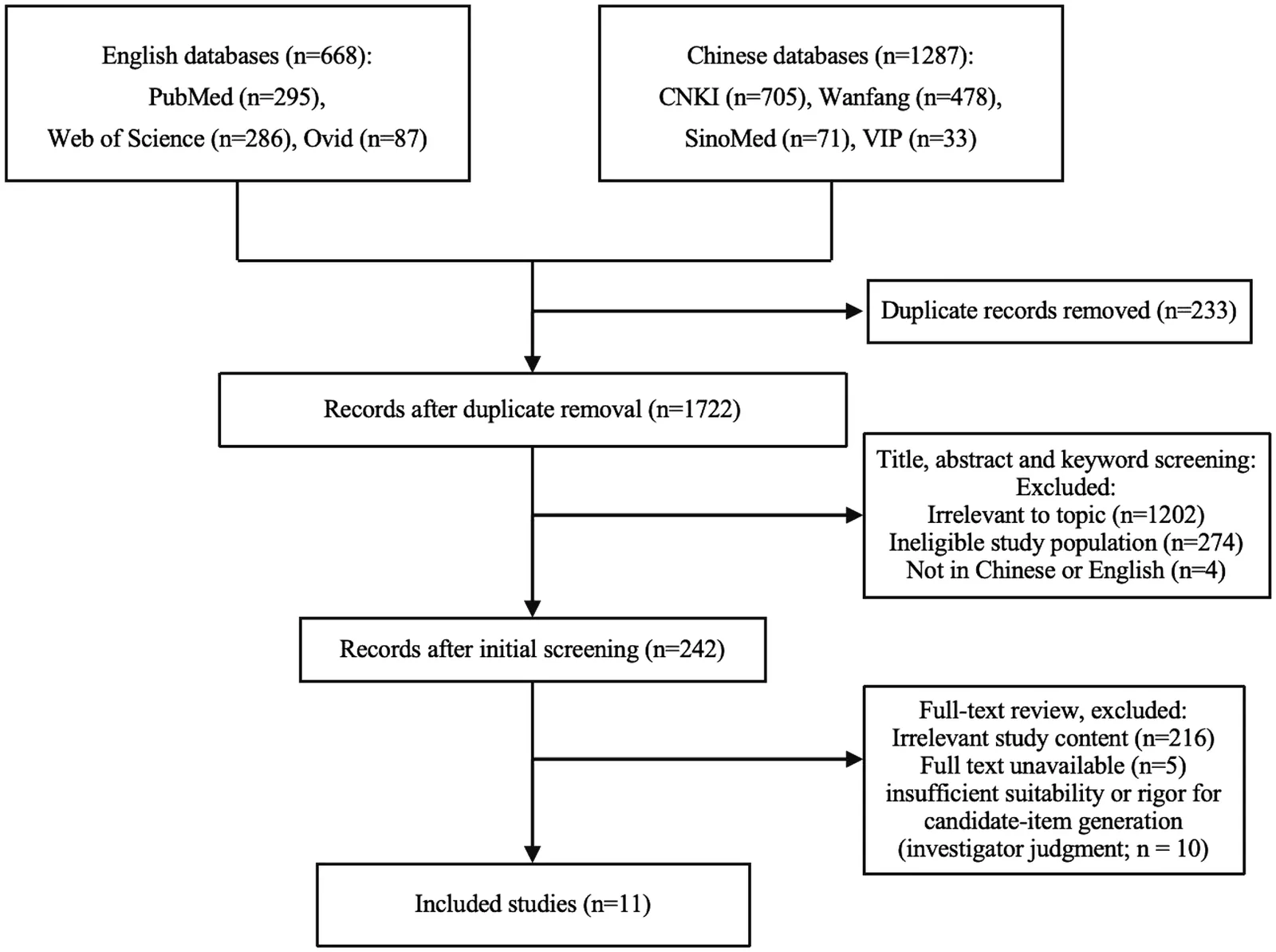
Literature screening process.

**Table 4:** Characteristics of the 11 included publications.

ID	Publication title and reference	Author(s)	Year	Evidence type
G1	Cancer-Associated Venous Thromboembolic Disease, Version 2.2024 (11)	Streiff et al.	2024	Guideline
G2	SEOM clinical guideline of venous thromboembolism (VTE) and cancer (2019) (12)	Muñoz Martín et al.	2020	Guideline
G3	American Society of Hematology 2021 guidelines for management of venous thromboembolism: prevention and treatment in patients with cancer (4)	Lyman et al.	2021	Guideline
G4	2022 international clinical practice guidelines for the treatment and prophylaxis of venous thromboembolism in patients with cancer, including patients with COVID-19 (5)	Farge et al.	2022	Guideline
G5	Venous thromboembolism in cancer patients: ESMO Clinical Practice Guideline (13)	Falanga et al.	2023	Guideline
G6	Venous Thromboembolism Prophylaxis and Treatment in Patients With Cancer: ASCO Guideline Update (14)	Key et al.	2023	Guideline
G7	Update on Guidelines for the Prevention of Cancer-Associated Thrombosis (15)	Elshoury et al.	2022	Review
G8	2021 Chinese guideline on the selection of anticoagulant drugs and pharmaceutical care for the prevention and treatment of venous thromboembolism (16)	Wang et al.	2021	Guideline
G9	Guidelines for the prevention and treatment of cancer-associated venous thromboembolism (2019 edition) (17)	CSCO Committee on Cancer and Thrombosis	2019	Guideline
G10	Guideline for quality evaluation and management of venous thromboembolism prevention and control in hospitals (2022 edition) (18)	National VTE project expert committee and guideline writing group	2022	Guideline
G11	Chinese expert consensus on mechanical prophylaxis for venous thromboembolism (19)	China Health Promotion Foundation expert committee	2020	Consensus

### Qualitative findings

Sixteen healthcare professionals participated in the interviews, including 9 physicians, 3 nurses, 2 clinical pharmacists, and 2 hospital administrators, and their characteristics were presented in Table 1.

As shown in Table 5, analysis of the 16 interviews identified facilitators in 7 CFIR constructs and barriers in 15 CFIR constructs, organized according to the five domains of the 2009 CFIR. Key facilitators included the perceived affordability of prophylactic medications, supportive policies and guidelines, leadership engagement, accessible training opportunities, and clinicians' positive attitudes toward prevention. Major barriers included the complexity of managing medication adherence and monitoring drug interactions and adverse reactions, limited patient understanding, gaps in clinicians' knowledge, unclear professional responsibilities, heavy workloads, and insufficient training and supervision. Participants also highlighted the absence of standardized prevention workflows and inadequate integration and feedback of outcome data. Illustrative participant quotations supporting these findings are provided in Supplementary Table S1.

**Table 5:** Facilitators and barriers to the implementation of VTE prevention in patients with lung cancer.

CFIR domain	CFIR construct	Barrier	Facilitator
Intervention characteristics	Complexity	Complexity of managing patients' medication adherence Complexity of monitoring drug interactions and adverse reactions	/
	Design quality and packaging	Lack of a lung cancer-specific assessment tool	/
	Cost	/	Low cost of prophylactic medications
Inner setting	Relative priority	Physicians primarily focus on the core clinical problem and tend to prioritize antitumor treatment VTE prevention has a low priority within the organization's internal management system	/
	Organizational incentives and rewards	Negative emotions caused by punitive measures The intensity of rewards and penalties affects physicians' willingness to implement prevention	Intrinsic motivation driven by research projects
	Structural characteristics	Unclear roles and responsibilities for VTE prevention within the organization Turnover of interns, postgraduate trainees, and rotating physicians affects implementation	/
	Tension for change	/	Adverse VTE outcomes had occurred in the hospital, increasing patients' medical costs
	Available resources	Heavy clinical workload, insufficient time, staff overload, and lack of dedicated personnel for the management and follow-up of patients receiving VTE prevention	/
	Compatibility	Poor compatibility with existing workflow	/
	Networks and communications	Lack of effective feedback and follow-up action after communication	/
	Access to knowledge and information	Insufficient training	High accessibility of training during departmental morning handovers
	Leadership engagement	Lack of supervision from leadership	Leadership showed a positive attitude after related medical events had occurred
Outer setting	External policy and incentives	System-level barriers caused by unclear authority and responsibility	National VTE-related guidelines and policies
	Patient needs and resources	Insufficient patient health literacy and low awareness of VTE	/
Characteristics of individuals	Knowledge and beliefs about the intervention	Lack of knowledge about cancer-related VTE risk assessment and management	/
	Self-efficacy	/	Physicians have a positive attitude toward prevention, believe that VTE prevention can provide health benefits to patients, and are willing to put it into practice
Process	Planning	Lack of standardized processes and unclear division of responsibilities in multidisciplinary collaboration	/
	Reflecting and evaluating	Lack of integration and feedback of outcome data, hindering data-driven continuous improvement	/

VTE, venous thromboembolism; CFIR, Consolidated Framework for Implementation Research.

### Candidate-item development

Integration of the evidence review and CFIR-based qualitative findings informed the development of the initial candidate-item pool. The evidence review contributed clinical prevention components, including risk assessment, general preventive care, mechanical and pharmacological prophylaxis, patient education, and outcome monitoring. The qualitative findings informed organizational and implementation components, including professional role allocation, staff training, workflow coordination, information support, and monitoring and feedback. Following research-team discussion and consolidation of overlapping content, the candidate items were organized with reference to Donabedian's SPO model. The first-round questionnaire comprised three overarching domains, 11 second-level components, and 38 candidate items. Detailed sources of the indicators at all levels were provided in Supplementary Table S2.

### Characteristics of the expert panel

Fifteen experts from Nanjing Drum Tower Hospital, a tertiary hospital in Jiangsu Province, China, participated in both Delphi rounds. The panel included experts in respiratory medicine (*n* = 4), medical oncology (*n* = 3), hospital administration (*n* = 2), clinical pharmacy (*n* = 3), and nursing administration (*n* = 3). One nursing expert had specific professional experience in hematology. The mean age of the experts was 41.87 ± 5.42 years, and their mean duration of professional experience was 16.67 ± 6.43 years (mean ± SD). Twelve experts held a master's or doctoral degree, and 11 held senior professional titles; the remaining 4 held intermediate professional titles (Table 6).

**Table 6:** Characteristics of the Delphi expert panel (*N* = 15).

Characteristic	Category	n or mea*n* ± SD	Percentage (%)
Gender	Male	7	46.7
	Female	8	53.3
Age (years)	Mean ± SD	41.87 ± 5.42	–
	< 40	7	46.7
	40–49	6	40.0
	50–59	2	13.3
Work experience (years)	Mean ± SD	16.67 ± 6.43	–
	10–19	10	66.7
	20–29	4	26.7
	≥ 30	1	6.7
Educational background	Bachelor's degree	3	20.0
	Master's degree	6	40.0
	Doctoral degree	6	40.0
Professional title	Intermediate title	4	26.7
	Associate senior title	7	46.7
	Senior title	4	26.7
Specialty	Respiratory medicine	4	26.7
	Medical oncology	3	20.0
	Hospital administration	2	13.3
	Clinical pharmacy	3	20.0
	Nursing administration	3	20.0

SD, standard deviation. Percentages are rounded to one decimal place and may not sum to 100%.

### Expert engagement and authority

All 15 initially invited experts agreed to participate and returned valid questionnaires in both rounds, giving a response rate of 100% in each round. The expert authority coefficient was 0.943 in round 1 and 0.953 in round 2 (Table 7).

**Table 7:** Expert authority coefficients in the two Delphi rounds.

Round	Judgment basis coefficient (Ca)	Familiarity coefficient (Cs)	Authority coefficient (Cr)
First	0.967	0.920	0.943
Second	0.987	0.920	0.953

### Consensus across Delphi rounds

Kendall's W values ranged from 0.249 to 0.292 across rounds and rating dimensions. In round 1, Kendall's W was 0.292 for importance and 0.250 for feasibility. In round 2, the corresponding values were 0.249 and 0.277. Although all tests were statistically significant (*P* < 0.001), the W values indicated limited overall concordance among experts (Table 8). Changes in W were not interpreted as improvement or deterioration in agreement because the item set and wording differed between rounds.

**Table 8:** Concordance among experts in the two Delphi rounds.

Round	Rating dimension	Experts (*n*)	Kendall's W	*χ*²	*P*
First	Importance	15	0.292	223.63	<0.001
	Feasibility	15	0.250	191.21	<0.001
Second	Importance	15	0.249	175.75	<0.001
	Feasibility	15	0.277	195.61	<0.001

**Table 9A:** Structural capacity indications.

Indicator	Importance		Feasibility	
	Mean ± SD	CV	Mean ± SD	CV
A. Structural capacity	4.93 ± 0.258	0.052	4.73 ± 0.458	0.097
A1 Institutional governance and protocols	4.93 ± 0.258	0.052	4.73 ± 0.594	0.126
A1.1 Standardized institutional protocol for VTE prevention across the care pathway	4.87 ± 0.352	0.072	4.73 ± 0.594	0.126
A1.2 Integration of VTE prevention into hospital quality management and performance monitoring	4.93 ± 0.258	0.052	4.73 ± 0.458	0.097
A2 Multidisciplinary workforce and role allocation	4.80 ± 0.561	0.117	4.60 ± 0.737	0.160
A2.1 Establishment of a multidisciplinary VTE prevention team	4.67 ± 0.488	0.104	4.73 ± 0.594	0.126
A2.2 Clear definition of professional responsibilities within the VTE prevention pathway	4.93 ± 0.258	0.052	4.93 ± 0.258	0.052
A2.3 Regular staff training and competency assessment for VTE prevention	4.13 ± 0.834	0.202	3.93 ± 0.799	0.203
A3 Infrastructure and supporting resources	4.60 ± 0.632	0.137	4.67 ± 0.488	0.104
A3.1 Electronic risk assessment and clinical decision-support system embedded in health records	4.20 ± 0.862	0.205	3.93 ± 0.961	0.245
A3.2 Availability of evidence-based pharmacological prophylaxis options	4.93 ± 0.258	0.052	4.93 ± 0.258	0.052
A3.3 Availability of mechanical prophylaxis devices	4.93 ± 0.258	0.052	4.93 ± 0.258	0.052
A3.4 Patient education materials in written and video formats	4.93 ± 0.258	0.052	4.93 ± 0.258	0.052

**Table 9B:** Care process f indications.

Indicator	Importance		Feasibility	
	Mean ± SD	CV	Mean ± SD	CV
B. Care processes	4.87 ± 0.352	0.072	4.60 ± 0.507	0.110
B1 Risk assessment and early recognition	4.73 ± 0.594	0.126	4.73 ± 0.458	0.097
B1.1 Timely assessment and reassessment of thrombosis and bleeding risks	4.93 ± 0.258	0.052	4.93 ± 0.258	0.052
B1.2 Use of validated tools for thrombosis and bleeding risk assessment (consider the Padua Prediction Score and the IMPROVE Bleeding Risk Score)	4.87 ± 0.352	0.072	4.87 ± 0.352	0.072
B1.3 Standardized diagnostic referral pathway for suspected lower-limb DVT	4.00 ± 0.756	0.189	4.20 ± 0.676	0.161
B1.4 Standardized escalation pathway for suspected pulmonary embolism	4.93 ± 0.258	0.052	4.93 ± 0.258	0.052
B1.5 Bedside prevention alert and daily handover verification for high-risk patients	4.93 ± 0.258	0.052	4.93 ± 0.258	0.052
B2 General preventive care	4.80 ± 0.414	0.086	4.13 ± 0.834	0.202
B2.1 Daily lower-limb assessment and patient reporting of warning signs	4.93 ± 0.258	0.052	4.93 ± 0.258	0.052
B2.2 Postural management and early mobilization support	4.93 ± 0.258	0.052	4.93 ± 0.258	0.052
B3 Mechanical prophylaxis	4.87 ± 0.352	0.072	4.73 ± 0.594	0.126
B3.1 Patient guidance on the appropriate use of compression stockings	4.93 ± 0.258	0.052	4.93 ± 0.258	0.052
B3.2 Screening for contraindications before mechanical prophylaxis	4.87 ± 0.352	0.072	4.87 ± 0.352	0.072
B3.3 Individualized mechanical prophylaxis plan and monitoring of actual use	4.13 ± 0.990	0.240	4.40 ± 0.828	0.188
B4 Medication safety in pharmacological prophylaxis	4.47 ± 0.640	0.143	4.07 ± 1.033	0.254
B4.1 Pre-prophylaxis assessment of thrombosis risk, bleeding risk, and medication history	4.93 ± 0.258	0.052	4.87 ± 0.352	0.072
B4.2 Multidisciplinary review and optimization of evidence-based pharmacological prophylaxis	4.13 ± 0.834	0.202	4.07 ± 0.884	0.217
B4.3 Standardized nursing procedures for anticoagulant administration	4.87 ± 0.352	0.072	4.93 ± 0.258	0.052
B5 Patient education and continuity of care	4.87 ± 0.352	0.072	4.67 ± 0.617	0.132
B5.1 Admission-based patient education on VTE prevention and treatment safety	4.87 ± 0.352	0.072	4.93 ± 0.258	0.052
B5.2 Individualized lifestyle and nutritional education: provide guidance on healthy lifestyle and appropriate nutritional support according to the patient's oncological and nutritional needs.	4.93 ± 0.258	0.052	4.93 ± 0.258	0.052
B5.3 Discharge medication safety counseling for patients receiving anticoagulant prophylaxis	4.87 ± 0.352	0.072	4.93 ± 0.258	0.052
B5.4 Structured post-discharge follow-up system for medication safety and self-monitoring	4.07 ± 0.961	0.236	3.60 ± 0.828	0.230

**Table 9C:** Outcome monitoring indications.

Indicator	Importance		Feasibility	
	Mean ± SD	CV	Mean ± SD	CV
C. Outcome monitoring	4.87 ± 0.352	0.072	4.93 ± 0.258	0.052
C1 Assessment quality indicators	4.87 ± 0.352	0.072	4.73 ± 0.458	0.097
C1.1 VTE risk assessment rate	4.93 ± 0.258	0.052	4.93 ± 0.258	0.052
C1.2 Bleeding risk assessment rate	4.93 ± 0.258	0.052	4.93 ± 0.258	0.052
C2 Prophylaxis implementation indicators	4.87 ± 0.352	0.072	4.73 ± 0.458	0.097
C2.1 Pharmacological prophylaxis implementation rate	4.93 ± 0.258	0.052	4.93 ± 0.258	0.052
C2.2 Mechanical prophylaxis implementation rate	4.93 ± 0.258	0.052	4.93 ± 0.258	0.052
C2.3 Combined prophylaxis implementation rate	4.93 ± 0.258	0.052	4.93 ± 0.258	0.052
C3 Clinical outcome indicators	4.80 ± 0.414	0.086	4.73 ± 0.458	0.097
C3.1 Incidence of VTE	4.93 ± 0.258	0.052	4.80 ± 0.414	0.086
C3.2 Incidence of bleeding events	4.80 ± 0.561	0.117	4.80 ± 0.561	0.117
C3.3 In-hospital VTE-related mortality	4.87 ± 0.352	0.072	4.93 ± 0.258	0.052

Scores are presented as mean ± SD. CV, coefficient of variation; VTE, venous thromboembolism; DVT, deep vein thrombosis. Prophylaxis implementation rates should be interpreted according to modality-specific eligibility and clinical appropriateness; higher rates do not necessarily indicate better care.

### Refinement of indicators across rounds

The initial questionnaire contained 38 third-level candidate indicators under 3 first-level domains and 11 second-level components. After round 1, quantitative ratings and qualitative feedback were reviewed. One indicator was deleted, six indicators were merged, and 12 indicators were revised to improve clarity, reduce overlap, and align the wording with service-delivery and implementation requirements. The round 2 questionnaire contained 34 third-level indicators. After round 2, all retained indicators were confirmed by the expert panel.

Three retained third-level indicators had round-2 mean feasibility scores below 4.00: staff training and competency assessment (A2.3, 3.93), electronic risk assessment and decision support (A3.1, 3.93), and structured post-discharge follow-up (B5.4, 3.60). Following research-team review of their clinical relevance and experts' written comments, these indicators were retained as implementation priorities despite not meeting the feasibility threshold. Item-level ratings, experts' written comments, revision decisions, and revised wording are provided in Supplementary Table S3. The original questionnaires used in the first and second Delphi rounds are provided in Supplementary Questionnaires S1 and S2, respectively.

### Final framework

The final framework comprised 3 first-level domains, 11 s-level components, and 34 third-level indicators (Table 9). The first-level domains were structural capacity, care processes, and outcome monitoring, corresponding to the three dimensions of the SPO model. Structural indicators covered institutional governance, multidisciplinary workforce allocation, and supporting resources. Process indicators covered risk assessment, general preventive care, mechanical prophylaxis, medication safety in pharmacological prophylaxis, and patient education and continuity of care. Outcome indicators covered assessment quality, prophylaxis implementation, and clinical outcomes.

### Operational specifications for monitoring measures

Provisional operational definitions, calculation formulas, and observation windows for the eight monitoring measures (C1.1–C3.3) are summarized in Table 10. The measures cover risk-assessment completion, appropriate prophylaxis among patients eligible for each modality, and in-hospital VTE, bleeding, and VTE-related mortality. Combined prophylaxis is not a universal target for medical inpatients. Detailed eligibility rules, exclusions, data sources, and ascertainment procedures are provided in Supplementary Table S4.

**Table 10:** Provisional operational definitions of the eight monitoring measures.

Framework code and measure	Operational definition	Calculation (%)	Observation window
C1.1 VTE risk assessment rate	Proportion of framework-cohort admissions with documented completion of VTE risk assessment within the specified window, including the tool, score, risk category and assessment time. For stays shorter than 24 h, completion is assessed before discharge.	$\frac{\begin{array}{c} \mathrm{Number}\;\mathrm{of}\;\mathrm{admissions}\;\mathrm{with}\;\mathrm{documented}\;\mathrm{VTE}\\ \mathrm{assessment}\;\mathrm{within}\;\mathrm{the}\;\mathrm{specified}\;\mathrm{window} \end{array}}{\begin{array}{c} \mathrm{Number}\;\mathrm{of}\;\mathrm{all}\;\mathrm{admissions}\;\;\mathrm{in}\;\mathrm{the}\;\mathrm{framework}\\ \mathrm{cohort} \end{array}}\times \;100\text{\%}$	Within 24 h of admission
C1.2 Bleeding risk assessment rate	Proportion of admissions with intermediate/high VTE risk under the source-defined eligibility rule that have a completed, documented bleeding-risk assessment within 24 h of admission. Unknown eligibility is reported separately.	$\frac{\begin{array}{c} \mathrm{Number}\;\mathrm{of}\;\mathrm{eligible}\;\mathrm{admissions}\;\mathrm{with}\;\mathrm{bleeding}\\ \mathrm{assessment}\;\mathrm{within}\;24\;h \end{array}}{\begin{array}{c} \mathrm{Number}\;\mathrm{of}\;\mathrm{admissions}\;\mathrm{with}\;\mathrm{source}\text{-}\mathrm{defined}\\ \mathrm{intermediate}/\mathrm{high}\;\mathrm{VTE}\;\mathrm{risk} \end{array}}\times 100\text{\%}$	Within 24 h of admission
C2.1 Pharmacological prophylaxis implementation rate	Proportion of admissions eligible for pharmacological primary prophylaxis that receive actual administration concordant with the specified guideline-based protocol, including agent, dose, route and timing during eligible intervals. Therapeutic-anticoagulation intervals and relevant contraindications are excluded.	$\frac{\begin{array}{c} \mathrm{Number}\;\mathrm{of}\;\mathrm{eligible}\;\mathrm{admissions}\;\mathrm{receiving}\\ \mathrm{guideline}\text{-}\mathrm{concordant}\;\mathrm{pharmacological}\\ \mathrm{prophylaxis} \end{array}}{\begin{array}{c} \mathrm{Number}\;\mathrm{of}\;\mathrm{admissions}\;\mathrm{eligible}\;\mathrm{for}\\ \mathrm{pharmacological}\;\mathrm{primary}\;\mathrm{prophylaxis} \end{array}}\times 100\text{\%}$	Eligible intervals during hospitalization
C2.2 Mechanical prophylaxis implementation rate	Proportion of admissions with a documented indication for mechanical prophylaxis and no relevant device-specific contraindication that receive the appropriate device with protocol-concordant application and duration. A prescription alone does not count as delivery.	$\frac{\begin{array}{c} \mathrm{Number}\;\mathrm{of}\;\mathrm{eligible}\;\mathrm{admissions}\;\mathrm{receiving}\\ \mathrm{guideline}\text{-}\mathrm{concordant}\;\mathrm{mechanical}\;\mathrm{prophylaxis} \end{array}}{\begin{array}{c} \mathrm{Number}\;\mathrm{of}\;\mathrm{admissions}\;\mathrm{eligible}\;\mathrm{for}\;\mathrm{mechanical}\\ \mathrm{prophylaxis} \end{array}}\times 100\text{\%}$	Eligible intervals during hospitalization
C2.3 Combined prophylaxis implementation rate	Proportion of admissions with an explicit indication for combined prophylaxis and no relevant contraindication to either modality that receive both appropriately within the same planned course. Sequential switching alone does not qualify; combination is not a universal target.	$\frac{\begin{array}{c} \mathrm{Number}\;\mathrm{of}\;\mathrm{eligible}\;\mathrm{admissions}\;\mathrm{receiving}\\ \mathrm{guideline}\text{-}\mathrm{concordant}\;\mathrm{combined}\;\mathrm{prophylaxis} \end{array}}{\begin{array}{c} \mathrm{Number}\;\mathrm{of}\;\mathrm{admissions}\;\mathrm{eligible}\;\mathrm{for}\;\mathrm{combined}\\ \mathrm{prophylaxis} \end{array}}\times 100\text{\%}$	Eligible intervals during hospitalization
C3.1 Incidence of VTE	Proportion of admissions without active VTE on admission in which newly occurring DVT or PE is objectively confirmed during hospitalization. Symptomatic and incidentally detected events are recorded separately. Events present on admission or occurring after discharge are excluded.	$\frac{\begin{array}{c} \mathrm{Number}\;\mathrm{of}\;\mathrm{eligible}\;\mathrm{admissions}\;\mathrm{with}\;\mathrm{new}\\ \mathrm{objectively}\;\mathrm{confirmed}\;\mathrm{hospital}\text{-}\mathrm{acquired}\;\mathrm{DVT}\\ \mathrm{or}\;\mathrm{PE} \end{array}}{\begin{array}{c} \mathrm{Number}\;\mathrm{of}\;\mathrm{framework}\text{-}\mathrm{cohort}\;\mathrm{admissions}\;\mathrm{without}\\ \mathrm{active}\;\mathrm{VTE}\;\mathrm{on}\;\mathrm{admission} \end{array}}\;\times 100\text{\%}$	Index hospitalization
C3.2 Incidence of bleeding events	Proportion of prophylactic-anticoagulation-exposed admissions with new ISTH major or clinically relevant non-major bleeding within the defined post-exposure window. Severity categories are reported separately; any subsequent therapeutic-anticoagulation exposure is identified.	$\frac{\begin{array}{c} \mathrm{Number}\;\mathrm{of}\;\mathrm{exposed}\;\mathrm{admissions}\;\mathrm{with}\;\mathrm{new}\\ \mathrm{qualifying}\;\mathrm{bleeding} \end{array}}{\begin{array}{c} \mathrm{Number}\;\mathrm{of}\;\mathrm{admissions}\;\mathrm{receiving}\;\mathrm{prophylactic}\\ \mathrm{anticoagulation} \end{array}}\times 100\text{\%}$	First prophylactic dose to discharge or in-hospital death
C3.3 In-hospital VTE-related mortality	Proportion of the eligible admission cohort with in-hospital death adjudicated as primarily or importantly attributable to objectively confirmed hospital-acquired VTE. The denominator includes eligible admissions without VTE events; this is not VTE case fatality.	$\frac{\begin{array}{c} \mathrm{Number}\;\mathrm{of}\;\mathrm{eligible}\;\mathrm{admissions}\;\mathrm{with}\\ \mathrm{adjudicated}\;\mathrm{hospital}\text{-}\mathrm{acquired}\text{-}\mathrm{VTE}\text{-}\mathrm{related}\\ \mathrm{death} \end{array}}{\begin{array}{c} \mathrm{Number}\;\mathrm{of}\;\mathrm{the}\;\mathrm{eligible}\;\mathrm{admission}\;\mathrm{cohort}\;\mathrm{used}\\ \mathrm{for}\;C3.1 \end{array}}\times 100\text{\%}$	Index hospitalization

VTE, venous thromboembolism; DVT, deep vein thrombosis; PE, pulmonary embolism; ISTH, International Society on Thrombosis and Haemostasis. These provisional specifications were developed after the Delphi consultation and require prospective testing and external measurement validation.

The pharmacological, mechanical, and combined prophylaxis implementation rates are descriptive measures and should not be interpreted as universal “higher-is-better” targets. Each rate should be interpreted in relation to eligibility for the corresponding modality, considering the reason for admission, thrombosis and bleeding risks, contraindications, and existing anticoagulant treatment. Brief treatment-only admissions and patients receiving therapeutic anticoagulation require separate consideration when defining the population eligible for pharmacological primary prophylaxis. Mechanical and combined prophylaxis are not universal performance targets, and a contraindication to pharmacological prophylaxis does not automatically establish eligibility for a particular mechanical modality. Higher implementation rates alone do not demonstrate more appropriate care; interpretation should also consider potentially inappropriate prophylaxis and missed prophylaxis among eligible patients.

The additional specifications were not evaluated in the original Delphi consultation and require prospective testing and external measurement validation. Clinical appropriateness criteria must be specified in a guideline-based institutional protocol before measurement.

For future implementation, potential inpatient tools include the Padua Prediction Score (20) or the IMPROVE VTE risk model (21), together with the IMPROVE Bleeding Risk Score (22). Their applicability to hospitalized patients with lung cancer requires local evaluation. The Khorana score was developed for cancer outpatients receiving chemotherapy (23) and should not be treated as an interchangeable inpatient risk model. Proposed bleeding ascertainment uses the ISTH definitions of major bleeding (24) and clinically relevant non-major bleeding (25). These source-based definitions and the handling of subsequent therapeutic anticoagulation are specified in Supplementary Table S4.

### Importance and feasibility of key indicators

Mean importance and feasibility scores were generally high across the framework. However, feasibility scores were lower than importance scores for several implementation-dependent indicators, particularly those involving electronic risk assessment (A3.1), staff training (A2.3), structured post-discharge follow-up (B5.4), and multidisciplinary review and optimization of pharmacological prophylaxis (B4.2) (Table 9). These differences were reported as importance–feasibility gaps for subsequent interpretation in the Discussion.

## Discussion

### Principal findings

This study developed a preliminary, consensus-based multidisciplinary service-delivery framework for VTE prevention and management in hospitalized patients with lung cancer. Using the SPO model, a structured literature review, semi-structured stakeholder interviews, and a two-round Delphi process, the final framework included 3 first-level domains, 11 second-level components, and 34 third-level indicators. The framework shifts the focus from isolated clinical decision-making to the organizational conditions, care processes, and quality indicators needed for reliable hospital-based VTE prevention (3–7).

### What this framework adds to current practice

Current cancer-associated thrombosis guidelines provide evidence-based recommendations for risk assessment and prophylaxis, but they provide less detail on how hospitals should operationalize these recommendations in routine care (3–5). The present framework addresses this implementation gap by translating guideline recommendations into service components, role allocation, workflow indicators, and quality-monitoring measures. Its contribution is therefore not a new anticoagulant regimen, but an operational structure for embedding VTE prevention into multidisciplinary hospital practice.

### Importance–feasibility gaps as a health services signal

The importance–feasibility gaps observed in the Delphi ratings are a useful implementation signal. Indicators related to electronic risk assessment, staff competency, protocol optimization, and structured post-discharge follow-up were considered important but relatively less feasible. This pattern suggests that the main barriers may lie in organizational readiness, information system integration, staffing capacity, workflow redesign, and continuity-of-care infrastructure. Similar multilevel barriers have been reported in the implementation of cancer-associated thrombosis guidelines and evidence-based practice more broadly (6, 26, 27).

### Implications for quality improvement and implementation

For quality improvement, the framework can help hospitals prioritize interventions according to both value and operational readiness. Indicators with high importance and high feasibility may be suitable for early implementation through routine audit, staff training, supervision, and feedback cycles. Indicators with high importance but lower feasibility may require staged implementation, managerial support, digital infrastructure, and local adaptation. This distinction may be particularly relevant for hospitals seeking to move from guideline awareness to measurable and sustainable practice change.

### Relevance of multidisciplinary care and pharmacists’ roles

The framework also clarifies the role of clinical pharmacists within a broader multidisciplinary model. Pharmacists should not be interpreted as replacing physician decision-making or leading an isolated drug-treatment pathway. Instead, their role is best framed in terms of medication safety, anticoagulant appropriateness review, drug interaction screening, renal and hepatic dose considerations, patient counseling, and continuity-of-care support. This framing aligns pharmacist involvement with public health service delivery and care coordination while avoiding overstatement of pharmacist-led effectiveness, which was not tested in this study.

### Future research and implementation evaluation

The framework should be prospectively tested in routine hospital settings using implementation and effectiveness outcomes. Future studies should evaluate feasibility, acceptability, fidelity, adoption, sustainability, and transferability across different hospital levels. Clinical and service outcomes such as risk assessment completion, appropriate prophylaxis, bleeding events, VTE incidence, readmission, patient knowledge, and post-discharge follow-up completion should also be assessed. Comparative or before-and-after quality improvement designs may help determine whether the framework improves service delivery and patient outcomes.

### Strengths and limitations

This study has several strengths. First, the framework was informed by multiple evidence sources rather than expert opinion alone, including literature review, stakeholder interviews, and Delphi consensus. Second, the SPO model provided a clear conceptual structure linking organizational capacity, care processes, and outcome monitoring (7).

Several limitations should be acknowledged. First, the panel comprised 15 experts recruited from a single tertiary hospital (Nanjing Drum Tower Hospital, Jiangsu Province, China), limiting the diversity of institutional perspectives and the transferability of the framework to other settings. Shared departmental affiliations and overlap between interview participants and Delphi panelists may have reinforced local perspectives and reduced the breadth of independent stakeholder input. Second, patients and family caregivers were not included in the Delphi panel, and their priorities may therefore be incompletely represented. Third, although the Delphi process established expert consensus, the framework has not yet been prospectively implemented or externally validated. Therefore, this study cannot demonstrate reductions in VTE incidence, bleeding events, mortality, or healthcare costs. Fourth, ratings were anonymous to other panelists but identifiable to the research team. The absence of structured feedback between rounds limited opportunities for informed reconsideration, while face-to-face questionnaire administration may have introduced social-desirability bias. Fifth, several indicators with high perceived importance had lower feasibility, suggesting that local adaptation, resource allocation, and information system support will be necessary before full implementation. Sixth, the absence of a standardized quality-appraisal tool introduced subjectivity into literature selection for candidate-item generation and may have influenced the initial item pool.

## Conclusion

This study developed a preliminary, consensus-based multidisciplinary service-delivery framework for VTE prevention and management in hospitalized medical patients with lung cancer using a mixed-methods approach informed by the SPO model, stakeholder interviews, and Delphi consensus. The framework translates existing guideline recommendations into operational indicators spanning structural capacity, care processes, medication safety, patient education, continuity of care, and outcome monitoring. It should be regarded as a foundation for hospital-based quality improvement and future implementation research rather than as definitive evidence of improved clinical outcomes.

## Data Availability

The raw data supporting the conclusions of this article will be made available by the authors, without undue reservation.
