# Supplementary material for: Development of a multidisciplinary service-delivery framework for venous thromboembolism prevention in hospitalized medical patients with lung cancer: a mixed-methods modified Delphi study
Source: Front Cardiovasc Med. 2026 Oct 28;13:1935436. doi: 10.3389/fcvm.2026.1935436 (PMC13752002; doi:10.3389/fcvm.2026.1935436)
Supplement: Supplementary file 1 [file Table1.docx]

**Supplementary Table S1. Sources and representative quotations for the identified facilitators and barriers**

| **Category** | **Theme** | **Representative quotation** |
| --- | --- | --- |
| **Intervention Characteristics** | | |
| Facilitator | Cost | A5: These medications are not expensive; they are all quite cheap. For example, low-molecular-weight heparin costs only about RMB 10 per day, and the reimbursement rate is relatively high. |
| Barrier | Complexity of managing patients' medication adherence; complexity of monitoring drug–drug interactions and adverse reactions | A1: A notable difference between lung cancer and other malignant tumors is its tendency to invade pulmonary vessels, which increases the risk of hemoptysis. Therefore, when considering anticoagulants for VTE prevention in patients with lung cancer, we need to balance the benefits of anticoagulation against the potential risk of hemoptysis. A9: One of the greatest concerns during medication use is the patient's bleeding risk. When bleeding occurs, patients may not report it promptly. Many patients also have poor medication adherence. |
| Barrier | Lack of a lung cancer-specific assessment tool | A2: The hospital currently uses the Caprini risk assessment scale, but its application is mostly limited to surgical settings. Its validity in patients with lung cancer still needs to be optimized. This limitation in clinical applicability highlights the need for a comprehensive risk assessment system tailored to the specific needs of medical patients. A1: I suggest simplifying the scale and providing a clearer and more concise scale or assessment criteria for us to use. |
| **Outer Setting** | | |
| Facilitator | National VTE-related guidelines and policies | A7: VTE has received increasing attention. I remember that several years ago, national-level inspections were conducted at our hospital, so I think this was a nationwide initiative. There are now also guidelines and documents related to thrombosis prevention. |
| Barrier | System-level barriers related to unclear accountability | A3: If preventive measures are taken for a patient and a problem occurs, the patient may attribute the responsibility to you. This reflects a fairly typical concern in the current doctor–patient relationship in China. Another issue is that, as we are still a developing country, some patients have limited knowledge and understanding and may not fully understand the necessity of prevention. As you mentioned, why are surgeons more willing to provide prevention? In my view, surgical patients come to the hospital to have their disease cured through surgery. If a complication occurs during or after surgery and leads to death, patients and their families often attribute the responsibility to the hospital. In internal medicine, especially medical oncology, patients are often already in the advanced stage of disease. If pulmonary embolism occurs, it may be regarded as a complication of the tumor, and the responsibility is not directly attributed to the physician because the underlying cause is still the tumor itself. |
| Barrier | Insufficient patient health literacy and low awareness of VTE | A3: Although we emphasize the importance of weekly blood monitoring after chemotherapy, some patients living in rural areas have limited literacy and health awareness and therefore have difficulty following medical advice. A7: Patients do not understand the purpose of the medication and focus all of their attention on lung cancer. Once the medication runs out, they stop taking it. This is very common. As a result, we found that some patients' lower-extremity thrombosis was never adequately controlled. |
| **Inner Setting** | | |
| Facilitator | Intrinsic motivation driven by research projects | A3: Just as you said, for example, when we are conducting research, we want to move the research project forward, so we work on it seriously. I think that is feasible. |
| Facilitator | Adverse VTE outcomes have occurred in the hospital, increasing patients' medical costs | B2: Everyone actually pays quite a lot of attention to this now because incidents have occurred, including sudden deaths due to pulmonary embolism or similar situations. A9: A patient developed a severe pulmonary embolism during hospitalization and subsequently received emergency thrombolysis and interventional treatment. Although the patient survived after active treatment, the pulmonary embolism resulted in several days in the intensive care unit and substantial additional medical costs. The patient was relatively young, and the family subsequently developed some emotional distress and conflict related to the event. This incident prompted both the department and hospital management to reflect deeply on and attach greater importance to the prevention of deep vein thrombosis. |
| Facilitator | High accessibility of training during departmental morning handovers | A7: You need to promote this in every department so that everyone pays sufficient attention. For example, after the main ward round—after communicating with the department leadership—you can use the time when everyone is present to provide training. It is similar to an in-department lecture; everyone can listen together, and it is efficient. It should not be too long, around 20 minutes, and should be simple and clear, focusing on the most important points. People may already understand the basics, but any updated content, such as guidelines for the prevention and treatment of VTE in solid tumors, can be reviewed. |
| Facilitator | Leadership became more supportive after VTE-related medical events occurred | A9: The leadership is still actively advancing this work because our hospital has previously experienced VTE-related medical events. |
| Barrier | Low priority of VTE prevention within the organizational management system | A8: In principle, our medical system is still treatment-oriented rather than prevention-oriented. Our hospital can only manage one part—essentially only one-third—while prevention and later rehabilitation are not fully covered, and no one really pays attention to those parts. D2: We formally proposed to the hospital administration that VTE prevention management be institutionalized and that prevention quality and implementation rates be incorporated into departmental or individual performance assessments. The proposal was not adopted after discussion, mainly because the hospital believed that the existing performance assessment system already contained too many indicators, and adding more indicators could create practical challenges related to management costs, clinical burden, and the usefulness of the indicators. |
| Barrier | Negative emotions triggered by punitive measures; the strength of rewards and penalties affects physicians' willingness to implement prevention | A9: Reward mechanisms can indeed improve motivation to some extent, but punitive measures may sometimes have the opposite effect and even cause resistance. In fact, oncologists usually focus more on the diagnosis and treatment plan for the tumor itself and find it difficult to pay attention to all of these details throughout the process. A3: Rewards and penalties may help improve the current situation, but their effect often depends on the strength of the incentive. For example, if the monthly reward or penalty is only a few hundred yuan, some physicians may feel that this simply adds extra workload and may instead become resistant. |
| Barrier | Ambiguous roles and unclear responsibilities for VTE prevention within the organization | D1: From a management perspective, it needs to be clear which department is responsible for training and which department is specifically responsible for this work. Should the medical affairs department provide the training, should the nursing department do it, or should another department be responsible? This involves professional knowledge, and people who are not specialists in this area may not be able to explain it clearly. C1: If one day the nursing assessment identifies an incorrect score, who should be responsible? This reflects the fact that responsibility remains unclear. There are still major gaps in defining who is responsible and what responsibility they should bear within the organizational structure, division of responsibilities, and implementation of formal policies. At present, different parties tend to work separately, and the nursing department is also reluctant to assume responsibility. |
| Barrier | Heavy clinical workload, insufficient time, and staff overload | A7: Clinical staff turnover is relatively high, while physician resources remain limited. It would be better to establish a system or designate dedicated personnel to conduct centralized risk assessment and remind physicians to provide prevention for patients. A5: Newly recruited interns, graduate students, and rotating physicians may lack sufficient knowledge or skills in VTE prevention, which can easily lead to gaps in the continuity of prevention work. A9: Outpatient physicians see a large number of patients each day, making it difficult to track whether preventive measures have been implemented for every patient. It is indeed difficult for physicians alone to manage all of these details because we face a very large number of patients. |
| Barrier | Poor compatibility with existing workflows | B1: If we talk about staff shortages, nurses may feel that this work will definitely take extra time. Overall, however, each procedure does not take very long. The problem is that, at exactly the time when the procedure needs to be performed—for example, when a pneumatic compression device should be applied or removed—the nurse may be busy with other tasks and therefore cannot complete it at the ideal time. The nurse may be receiving a new patient or providing treatment such as an infusion. They may decide to finish the current task first, but by the time they return, half an hour may already have passed. |
| Barrier | Lack of effective feedback and follow-up action after communication | B1: For example, a nurse may notice a risk and communicate it to the physician. Because the physician is very busy, suggestions such as whether the patient needs a lower-extremity vascular ultrasound to rule out thrombosis or whether pneumatic compression can be used may not be addressed or adopted immediately. Sometimes the physician agrees that there is a problem, but after the nurse raises it several times and feels that there has still been no follow-up, the nurse may worry that repeatedly bringing it up will annoy the physician. |
| Barrier | Insufficient training | A1: I do not think there has been much training; over the years, I have only encountered it a few times. For rivaroxaban, for example, what dose should be used to prevent thrombosis while reducing bleeding risk? We previously gave patients 10 mg, but the effect did not seem very satisfactory. |
| Barrier | Lack of leadership supervision | A8: No leader has specifically discussed this with me, and it is still not given much attention. I know that we have a VTE prevention and treatment group and that pop-up reminders appear in the system, but no one has specifically emphasized this issue. |
| **Characteristics of Individuals** | | |
| Facilitator | Physicians hold positive attitudes toward prevention, believe that VTE prevention can benefit patients, and are willing to put it into practice | A4: I am positive about it. I think it is a good thing because some patients with cancer do develop thrombotic events, which are unfavorable and also affect their prognosis. A9: I think the key is to provide sufficient education to clinical staff. Physicians must truly understand the importance of this work before the measures can be implemented in clinical practice. Otherwise, even with a good plan, it is still the frontline physicians who directly interact with patients who ultimately have to carry it out at the bedside. |
| Barrier | Knowledge gaps in cancer-associated VTE risk assessment and management | A1: Many clinicians are not familiar with these risk assessment scales, and I feel that my own understanding of the guidelines is not sufficiently thorough. I think this part of treatment is still very challenging. What is the patient's liver or kidney function? How should the medication be adjusted? Particularly for direct oral anticoagulants, there are differences among drugs. The types of drugs differ, and the duration of anticoagulant treatment also varies. |
| **Process** | | |
| Barrier | Lack of standardized procedures and unclear division of responsibilities in multidisciplinary collaboration | A9: Our department has not yet established a systematic VTE prevention implementation process. This may be an important area for improvement. A7: I believe that pharmacists and nurses, together with multidisciplinary collaboration, can play a better role in VTE prevention. Physicians usually focus more on treatment of the disease itself, whereas the specific implementation of prevention often requires key involvement from pharmacists and nurses... In practice, we sometimes encounter special situations, such as an older patient with cancer whose D-dimer level is markedly elevated. We would like to provide anticoagulant prophylaxis, but because of the patient's advanced age, we hesitate to use low-molecular-weight heparin or oral anticoagulants. In such cases, pharmacists are exactly the professionals who can provide guidance on medication use and risk assessment. |
| Barrier | Lack of integrated outcome data and feedback, hindering data-driven continuous improvement | D1: Data feedback must first be based on clearly defined indicators, such as the prevention rate or the extent to which the incidence of thrombosis has decreased. Once these indicators are defined, the relevant administrative departments need to provide regular feedback to clinical departments, for example through weekly or monthly reports. This is indeed something that administrative departments should be responsible for. For data extraction, support from the information center is needed. They need to be told clearly what data should be extracted and where it should be extracted from—for example, whether from the HIS or the nursing documentation system. The data source and extraction pathway must be clearly defined before the data can actually be retrieved. A1: After patients receive low-molecular-weight heparin or similar medications, what is their bleeding risk? Have they ever stopped the medication because of bleeding? How often are patients reassessed after starting medication? We need to assess whether the risk of thrombosis has decreased and whether there is a risk of bleeding. A2: We need to follow patients through surveys and data integration and compare whether the incidence of thrombosis decreases after they receive VTE preventive treatment. |

*Note.* Quotations were translated from Chinese into English for publication while preserving the original meaning. VTE, venous thromboembolism; HIS, hospital information system.

**Supplementary Table S2. Draft VTE Prevention Management Strategy for Patients with Lung Cancer**

| **Level 1 indicator** | **Source** | **Level 2 indicator** | **Source** | **Level 3 indicator** | **Source** |
| --- | --- | --- | --- | --- | --- |
| A. Structure indicators | Structure–Process–Outcome model | A1. Policies and procedures | Qualitative research findings | A1.1 Develop and implement, through team collaboration, a standardized VTE prevention management process covering the entire treatment course of patients with lung cancer. The process should include specifications for risk assessment, prevention decision-making, medication implementation and monitoring, patient education, and emergency management. The team leader should supervise process implementation, develop improvement plans, and carry out continuous quality improvement. | Qualitative research findings |
| A. Structure indicators | Structure–Process–Outcome model | A1. Policies and procedures | Qualitative research findings | A1.2 Incorporate VTE prevention management into the hospital quality management system and performance assessment indicators. Establish unified policies for the prevention and management of lung cancer-associated VTE, regularly evaluate the implementation of preventive measures in relevant departments, and provide timely feedback and improvement. | Qualitative research findings |
| A. Structure indicators | Structure–Process–Outcome model | A2. Human resources | Qualitative research findings | A2.1 Establish a multidisciplinary VTE prevention team. Core members should include physicians from respiratory and critical care medicine/medical oncology, vascular surgeons (as appropriate), clinical pharmacists, and nurses from respiratory medicine/medical oncology. | Literature review and qualitative research findings |
| A. Structure indicators | Structure–Process–Outcome model | A2. Human resources | Qualitative research findings | A2.2 Define the specific responsibilities of team members: (1) Physicians should play the central management role, be responsible for comprehensive patient assessment, assess VTE risk using tools such as the Khorana score/Padua score, integrate team recommendations, and formulate prevention plans according to patient factors; (2) Pharmacists should review the appropriateness of anticoagulant prophylaxis and optimize medication safety, with particular attention to bleeding tendency and drug–drug interactions, review individualized doses (especially for patients with hepatic or renal impairment), monitor adverse reactions, and participate in medication education and follow-up for anticoagulants; (3) Nurses should assist physicians with VTE risk assessment, implement preventive measures, provide basic prevention education, deliver individualized health education to patients and their families, and standardize documentation of the implementation of preventive measures and patient responses. | Qualitative research findings |
| A. Structure indicators | Structure–Process–Outcome model | A2. Human resources | Qualitative research findings | A2.3 Provide diversified training to improve healthcare professionals’ ability to screen and assess VTE risk, manage prevention, and provide health education. Regularly assess healthcare professionals’ knowledge and skills related to VTE prevention and continuously reinforce training according to the assessment results. | Qualitative research findings |
| A. Structure indicators | Structure–Process–Outcome model | A3. Material resources | Literature review findings | A3.1 Establish an electronic VTE risk assessment system (integrating thrombosis risk assessment tools such as the Khorana score/Padua score and bleeding risk assessment tools such as the IMPROVE score), and embed risk assessment into the electronic medical record to prompt healthcare professionals to intervene in a timely manner. | Qualitative research findings |
| A. Structure indicators | Structure–Process–Outcome model | A3. Material resources | Literature review findings | A3.2 Ensure an adequate supply of anticoagulants required for VTE prevention (e.g., low-molecular-weight heparin and rivaroxaban) to ensure medication accessibility. | Literature review findings |
| A. Structure indicators | Structure–Process–Outcome model | A3. Material resources | Literature review findings | A3.3 Provide necessary mechanical prophylaxis equipment (graduated compression stockings and intermittent pneumatic compression devices) to meet patients’ needs for mechanical prophylaxis. | Qualitative research findings |
| A. Structure indicators | Structure–Process–Outcome model | A3. Material resources | Literature review findings | A3.4 Educational materials: Provide patients with educational booklets on VTE prevention and management, and prepare corresponding video materials for patients and their families to ensure that they understand basic prevention knowledge. | Literature review findings |
| B. Process indicators | Structure–Process–Outcome model | B1. Risk assessment and stratification | Literature review and qualitative research findings | B1.1 Assess the risk of thrombosis and bleeding within 24 hours of admission. If changes in the patient’s condition may alter the thrombosis risk level, reassessment should be performed promptly. | Literature review findings |
| B. Process indicators | Structure–Process–Outcome model | B1. Risk assessment and stratification | Literature review and qualitative research findings | B1.2 Assessment tools: The Khorana score/Padua score is recommended for assessing thrombosis risk, and the IMPROVE bleeding risk score is recommended for assessing bleeding risk. | Literature review, literature review findings |
| B. Process indicators | Structure–Process–Outcome model | B1. Risk assessment and stratification | Literature review and qualitative research findings | B1.3 For patients with suspected lower-extremity deep vein thrombosis (DVT), lower-extremity venous ultrasonography is the preferred imaging screening method. | Literature review findings |
| B. Process indicators | Structure–Process–Outcome model | B1. Risk assessment and stratification | Literature review and qualitative research findings | B1.4 D-dimer testing may assist in screening for acute pulmonary thromboembolism (PTE). When D-dimer is markedly elevated, pulmonary artery CT angiography or lower-extremity venous ultrasonography should be completed within 12 hours. | Literature review findings |
| B. Process indicators | Structure–Process–Outcome model | B1. Risk assessment and stratification | Literature review and qualitative research findings | B1.5 For hospitalized patients assessed as high risk, place a “thrombosis prevention warning sign” at the bedside and verify the implementation of preventive measures during the daily morning handover. | Literature review findings |
| B. Process indicators | Structure–Process–Outcome model | B2. Basic prevention | Literature review findings | B2.1 Fluid intake: When clinically appropriate, ensure an adequate daily fluid intake (approximately 1500–2500 mL of water; adjust as appropriate for patients with cardiac insufficiency). | Literature review findings |
| B. Process indicators | Structure–Process–Outcome model | B2. Basic prevention | Literature review findings | B2.2 Lifestyle modification: Provide guidance and support for smoking and alcohol cessation, assist patients in developing healthy lifestyle habits, formulate a low-salt, low-sugar, low-fat, high-protein diet plan, and supervise its implementation. | Literature review findings |
| B. Process indicators | Structure–Process–Outcome model | B2. Basic prevention | Literature review findings | B2.3 Monitor the lower extremities daily: Assess both lower extremities for swelling, changes in skin temperature, abnormal coloration, pain, and other abnormalities. If abnormalities are identified, report them to the healthcare team within 2 hours and manage them promptly. | Literature review findings |
| B. Process indicators | Structure–Process–Outcome model | B2. Basic prevention | Literature review findings | B2.4 Optimize positioning and early functional exercise: Elevate the lower extremities (20°–30°), avoid placing a hard pillow beneath the knees and excessive hip flexion to promote venous return; encourage patients to begin active/passive in-bed activities as early as possible, such as ankle-pump exercises and quadriceps contractions. | Literature review findings |
| B. Process indicators | Structure–Process–Outcome model | B3. Mechanical prophylaxis | Literature review findings | B3.1 Screen for contraindications before mechanical prophylaxis: Before using graduated compression stockings (GCS) or intermittent pneumatic compression (IPC) for mechanical prophylaxis, assess for contraindications such as peripheral arterial disease and skin damage. | Literature review findings |
| B. Process indicators | Structure–Process–Outcome model | B3. Mechanical prophylaxis | Literature review findings | B3.2 Suitability and management of mechanical prophylaxis: For patients at high risk of bleeding who are temporarily unable to receive pharmacological prophylaxis, mechanical prophylaxis alone (e.g., GCS or IPC) may be used. The regimen should be adjusted according to patient tolerance. IPC is recommended for a cumulative duration of at least 18 hours per day; for completely bedridden patients, use should be extended as much as possible to improve the preventive effect. Once bleeding risk decreases, patients should be reassessed promptly and switched to or combined with pharmacological prophylaxis. | Literature review findings |
| B. Process indicators | Structure–Process–Outcome model | B3. Mechanical prophylaxis | Literature review findings | B3.3 Care during mechanical prophylaxis: Assess skin integrity at the application site daily. If pressure marks, skin pallor, or pain occurs, discontinue mechanical prophylaxis immediately and document and manage the event accordingly. | Literature review findings |
| B. Process indicators | Structure–Process–Outcome model | B4. Pharmacological prophylaxis | Qualitative research findings | B4.1 Pre-medication assessment and decision-making: Physicians should comprehensively assess the patient’s VTE and bleeding risks and inquire about and document previous medical history (including thrombotic events, bleeding history, and medication history). For patients at high thrombosis risk and low bleeding risk, an individualized pharmacological prophylaxis plan should be developed, and informed consent should be obtained before medication initiation regarding the purpose and importance of prevention and potential adverse effects. | Qualitative research findings |
| B. Process indicators | Structure–Process–Outcome model | B4. Pharmacological prophylaxis | Qualitative research findings | B4.2 Optimization of medication selection and implementation of medical orders: For patients newly starting systemic chemotherapy who are at very high risk of thrombosis (Khorana score ≥2/Padua score ≥4), if there are no obvious high-risk bleeding factors or important drug–drug interactions, physicians should preferentially consider rivaroxaban or low-molecular-weight heparin for prophylactic anticoagulation and promptly issue the corresponding prophylaxis orders based on the assessment results. | Qualitative research findings |
| B. Process indicators | Structure–Process–Outcome model | B4. Pharmacological prophylaxis | Qualitative research findings | B4.3 Pharmacist prescription review: Clinical pharmacists should review anticoagulant prescriptions issued by physicians within 24 hours of admission (including medication type, dose, and treatment duration), optimize the anticoagulation regimen according to hepatic and renal function and other patient factors, and provide necessary recommendations for adjustment. | Qualitative research findings |
| B. Process indicators | Structure–Process–Outcome model | B4. Pharmacological prophylaxis | Qualitative research findings | B4.4 Nursing standards for medication administration: Nurses should strictly follow standardized procedures when injecting anticoagulants. Subcutaneous abdominal injection should be preferred for low-molecular-weight heparin, and prefilled low-molecular-weight heparin syringes should be used. Air should not be expelled before injection; the needle should be inserted slowly and vertically, and the injection site should not be compressed after needle withdrawal, to reduce bleeding risk. | Qualitative research findings |
| B. Process indicators | Structure–Process–Outcome model | B5. Health education and follow-up | Quantitative and qualitative research findings | B5.1 Admission education: On the day of admission, healthcare professionals should provide bedside health education, introduce VTE-related signs and symptoms, emphasize the importance of VTE prevention, and explain possible adverse effects of anticoagulant prophylaxis to improve patient adherence to prevention. | Qualitative research findings |
| B. Process indicators | Structure–Process–Outcome model | B5. Health education and follow-up | Quantitative and qualitative research findings | B5.2 Guidance on compression stockings: Instruct patients on the correct selection and use of graduated compression stockings for prevention, and emphasize the importance of continuous use and precautions (e.g., putting them on and taking them off at scheduled times each day and maintaining limb cleanliness). | Literature review findings |
| B. Process indicators | Structure–Process–Outcome model | B5. Health education and follow-up | Quantitative and qualitative research findings | B5.3 Dietary guidance: Provide appropriate dietary recommendations for hospitalization and after discharge, and instruct patients to maintain a low-salt, low-fat diet with balanced nutrition to reduce thrombosis risk. | Literature review findings |
| B. Process indicators | Structure–Process–Outcome model | B5. Health education and follow-up | Quantitative and qualitative research findings | B5.4 Discharge guidance: At discharge, clinical pharmacists should provide specific guidance on the correct use of anticoagulants, including dose, frequency, treatment duration, precautions, and possible adverse reactions, to ensure safe and standardized medication use after discharge. | Qualitative research findings |
| B. Process indicators | Structure–Process–Outcome model | B5. Health education and follow-up | Quantitative and qualitative research findings | B5.5 Establish a pharmaceutical care and follow-up system: Create individualized follow-up records and plans for patients receiving anticoagulant prophylaxis. Pharmacists should provide pharmaceutical care through regular telephone or outpatient follow-up, with particular attention to bleeding and other adverse reactions (e.g., examination of skin and mucosa, gums, conjunctiva, ecchymoses, and petechiae), changes in platelet count, and medication adherence; follow-up test results, VTE recurrence, and other long-term complications should also be documented to comprehensively evaluate preventive effectiveness and provide timely intervention and management. | Qualitative research findings |
| C. Outcome indicators | Structure–Process–Outcome model | C1. Assessment quality indicators | Literature review findings, qualitative research findings | C1.1 VTE risk assessment rate (proportion of hospitalized patients who undergo VTE risk assessment). | Qualitative research findings |
| C. Outcome indicators | Structure–Process–Outcome model | C1. Assessment quality indicators | Literature review findings, qualitative research findings | C1.2 Bleeding risk assessment rate (proportion of hospitalized patients who undergo bleeding risk assessment). | Qualitative research findings |
| C. Outcome indicators | Structure–Process–Outcome model | C2. Prevention quality indicators | Literature review findings, qualitative research findings | C2.1 Pharmacological prophylaxis implementation rate (proportion of patients with indications for pharmacological prophylaxis who actually receive anticoagulant prophylaxis). | Qualitative research findings |
| C. Outcome indicators | Structure–Process–Outcome model | C2. Prevention quality indicators | Literature review findings, qualitative research findings | C2.2 Mechanical prophylaxis implementation rate (proportion of patients with indications for mechanical prophylaxis who actually receive mechanical prophylaxis). | Literature review findings |
| C. Outcome indicators | Structure–Process–Outcome model | C2. Prevention quality indicators | Literature review findings, qualitative research findings | C2.3 Combined prophylaxis implementation rate (proportion of patients with indications for combined prophylaxis who receive both pharmacological and mechanical prophylaxis). | Literature review findings |
| C. Outcome indicators | Structure–Process–Outcome model | C3. Outcome quality indicators | Literature review findings, qualitative research findings | C3.1 In-hospital VTE incidence (proportion of patients with lung cancer who experience a venous thromboembolic event during hospitalization). | Qualitative research findings |
| C. Outcome indicators | Structure–Process–Outcome model | C3. Outcome quality indicators | Literature review findings, qualitative research findings | C3.2 Incidence of bleeding events (proportion of patients who experience bleeding adverse events during prevention, including minor and major bleeding). | Literature review findings |
| C. Outcome indicators | Structure–Process–Outcome model | C3. Outcome quality indicators | Literature review findings, qualitative research findings | C3.3 Hospital-associated VTE mortality (proportion of patients who die due to hospital-acquired VTE). | Literature review findings |

**Supplementary Table S3. Round-1 Delphi audit trail for all item codes reported in the thesis**

| **Item** | **Level** | **Round-1 wording** | **Importance, mean±SD (CV)** | **Feasibility, mean±SD (CV)** | **Expert comment(s)** | **Decision after round 1** | **Round-2 wording/status** |
| --- | --- | --- | --- | --- | --- | --- | --- |
| A | Level 1 | Structure indicators | 4.87±0.352 (0.072) | 4.87±0.352 (0.072) | None | Retained without change. | Unchanged from round 1. |
| A1 | Level 2 | Policies and procedures | 4.87±0.352 (0.072) | 4.87±0.352 (0.072) | None | Retained without change. | Unchanged from round 1. |
| A1.1 | Level 3 | A1.1 Develop and implement, through team collaboration, a standardized VTE prevention management process covering the entire treatment course of patients with lung cancer. The process should include specifications for risk assessment, prevention decision-making, medication implementation and monitoring, patient education, and emergency management. The team leader should supervise process implementation, develop improvement plans, and carry out continuous quality improvement. | 4.87±0.352 (0.072) | 4.60±0.632 (0.137) | None | Retained without change. | Unchanged from round 1. |
| A1.2 | Level 3 | A1.2 Incorporate VTE prevention management into the hospital quality management system and performance assessment indicators. Establish unified policies for the prevention and management of lung cancer-associated VTE, regularly evaluate the implementation of preventive measures in relevant departments, and provide timely feedback and improvement. | 4.60±0.507 (0.110) | 4.27±0.704 (0.165) | None | Retained without change. | Unchanged from round 1. |
| A2 | Level 2 | Human resources | 4.80±0.414 (0.086) | 4.47±0.640 (0.143) | None | Retained without change. | Unchanged from round 1. |
| A2.1 | Level 3 | A2.1 Establish a multidisciplinary VTE prevention team. Core members should include physicians from respiratory and critical care medicine/medical oncology, vascular surgeons (as appropriate), clinical pharmacists, and nurses from respiratory medicine/medical oncology. | 4.80±0.414 (0.086) | 4.33±0.900 (0.208) | None | Retained without change. | Unchanged from round 1. |
| A2.2 | Level 3 | A2.2 Define the specific responsibilities of team members: (1) Physicians should play the central management role, be responsible for comprehensive patient assessment, assess VTE risk using tools such as the Khorana score/Padua score, integrate team recommendations, and formulate prevention plans according to patient factors; (2) Pharmacists should review the appropriateness of anticoagulant prophylaxis and optimize medication safety, with particular attention to bleeding tendency and drug–drug interactions, review individualized doses (especially for patients with hepatic or renal impairment), monitor adverse reactions, and participate in medication education and follow-up for anticoagulants; (3) Nurses should assist physicians with VTE risk assessment, implement preventive measures, provide basic prevention education, deliver individualized health education to patients and their families, and standardize documentation of the implementation of preventive measures and patient responses. | 4.87±0.352 (0.072) | 4.60±0.632 (0.137) | None | Retained without change. | Unchanged from round 1. |
| A2.3 | Level 3 | A2.3 Provide diversified training to improve healthcare professionals’ ability to screen and assess VTE risk, manage prevention, and provide health education. Regularly assess healthcare professionals’ knowledge and skills related to VTE prevention and continuously reinforce training according to the assessment results. | 4.80±0.414 (0.086) | 4.53±0.915 (0.202) | None | Retained without change. | Unchanged from round 1. |
| A3 | Level 2 | Material resources | 4.87±0.352 (0.072) | 4.53±0.640 (0.141) | None | Retained without change. | Unchanged from round 1. |
| A3.1 | Level 3 | A3.1 Establish an electronic VTE risk assessment system (integrating thrombosis risk assessment tools such as the Khorana score/Padua score and bleeding risk assessment tools such as the IMPROVE score), and embed risk assessment into the electronic medical record to prompt healthcare professionals to intervene in a timely manner. | 4.87±0.352 (0.072) | 4.60±0.632 (0.137) | None | Retained without change. | Unchanged from round 1. |
| A3.2 | Level 3 | A3.2 Ensure an adequate supply of anticoagulants required for VTE prevention (e.g., low-molecular-weight heparin and rivaroxaban) to ensure medication accessibility. | 4.73±0.458 (0.097) | 4.67±0.617 (0.132) | None | Retained without change. | Unchanged from round 1. |
| A3.3 | Level 3 | A3.3 Provide necessary mechanical prophylaxis equipment (graduated compression stockings and intermittent pneumatic compression devices) to meet patients’ needs for mechanical prophylaxis. | 4.93±0.258 (0.052) | 4.87±0.352 (0.072) | None | Retained without change. | Unchanged from round 1. |
| A3.4 | Level 3 | A3.4 Educational materials: Provide patients with educational booklets on VTE prevention and management, and prepare corresponding video materials for patients and their families to ensure that they understand basic prevention knowledge. | 4.67±0.488 (0.104) | 4.67±0.617 (0.132) | None | Retained without change. | Unchanged from round 1. |
| B | Level 1 | Process indicators | 4.87±0.352 (0.072) | 4.53±0.640 (0.141) | None | Retained without change. | Unchanged from round 1. |
| B1 | Level 2 | Risk assessment and stratification | 4.73±0.594 (0.126) | 4.60±0.737 (0.160) | None | Retained without change. | Unchanged from round 1. |
| B1.1 | Level 3 | B1.1 Assess the risk of thrombosis and bleeding within 24 hours of admission. If changes in the patient’s condition may alter the thrombosis risk level, reassessment should be performed promptly. | 4.00±0.756 (0.189) | 3.60±0.737 (0.205) | None | Retained without change. | Unchanged from round 1. |
| B1.2 | Level 3 | B1.2 Assessment tools: The Khorana score/Padua score is recommended for assessing thrombosis risk, and the IMPROVE bleeding risk score is recommended for assessing bleeding risk. | 3.87±0.743 (0.192) | 3.60±0.737 (0.205) | None | Retained without change. | Unchanged from round 1. |
| B1.3 | Level 3 | B1.3 For patients with suspected lower-extremity deep vein thrombosis (DVT), lower-extremity venous ultrasonography is the preferred imaging screening method. | 4.20±0.775 (0.185) | 3.87±0.834 (0.216) | None | Retained without change. | Unchanged from round 1. |
| B1.4 | Level 3 | B1.4 D-dimer testing may assist in screening for acute pulmonary thromboembolism (PTE). When D-dimer is markedly elevated, pulmonary artery CT angiography or lower-extremity venous ultrasonography should be completed within 12 hours. | 4.27±0.594 (0.139) | 4.00±0.756 (0.189) | The clinical operability of “complete pulmonary artery CT angiography or lower-extremity venous ultrasonography within 12 hours” was questioned. The expert recommended a more flexible implementation framework consistent with real-world resource conditions. | Modified; retained as B1.4 | B1.4 D-dimer testing may assist in screening for acute pulmonary thromboembolism (PTE). When D-dimer is markedly elevated, urgent management should be undertaken according to the clinical situation: for patients with high clinical suspicion of PTE, an emergency diagnostic pathway should be initiated and pulmonary artery CT angiography actively coordinated within 12 hours; for those with low-to-moderate suspicion, lower-extremity venous ultrasonography and other examinations should be arranged as soon as possible, with close monitoring for changes in condition. |
| B1.5 | Level 3 | B1.5 For hospitalized patients assessed as high risk, place a “thrombosis prevention warning sign” at the bedside and verify the implementation of preventive measures during the daily morning handover. | 4.47±0.834 (0.187) | 4.67±0.617 (0.132) | Recommended revision: identify hospitalized patients receiving thrombosis prevention and treatment with a bedside “thrombosis prevention warning sign,” and verify implementation of preventive measures during the daily morning handover. | Modified; retained as B1.5 | B1.5 For hospitalized patients receiving thrombosis prevention and treatment, place a “thrombosis prevention warning sign” at the bedside and verify implementation of preventive measures during the daily morning handover. |
| B2 | Level 2 | Basic prevention | 4.60±0.632 (0.137) | 4.20±0.862 (0.205) | None | Retained without change. | Unchanged from round 1. |
| B2.1 | Level 3 | B2.1 Fluid intake: When clinically appropriate, ensure an adequate daily fluid intake (approximately 1500–2500 mL of water; adjust as appropriate for patients with cardiac insufficiency). | 3.60±0.737 (0.205) | 3.53±0.915 (0.259) | None | Deleted after screening and research-team discussion | Deleted; no round-2 wording. |
| B2.2 | Level 3 | B2.2 Lifestyle modification: Provide guidance and support for smoking and alcohol cessation, assist patients in developing healthy lifestyle habits, formulate a low-salt, low-sugar, low-fat, high-protein diet plan, and supervise its implementation. | 4.07±0.704 (0.173) | 3.80±0.775 (0.204) | Overlaps with B5.3; consider merging the two. The terms “low-salt, low-sugar, low-fat, high-protein” were considered insufficiently defined. | Modified and merged with B5.3; represented as round-2 B5.2 | B5.2 Dietary guidance: Provide reasonable dietary advice during hospitalization and after discharge; instruct patients to maintain a low-salt, low-fat diet and balanced nutrition (e.g., daily salt intake <5 g, cooking oil 25–30 g, added sugar <25 g, and adequate high-quality protein such as fish, poultry, eggs, milk, and soy products) to reduce thrombosis risk. |
| B2.3 | Level 3 | B2.3 Monitor the lower extremities daily: Assess both lower extremities for swelling, changes in skin temperature, abnormal coloration, pain, and other abnormalities. If abnormalities are identified, report them to the healthcare team within 2 hours and manage them promptly. | 4.13±0.743 (0.180) | 3.60±0.828 (0.230) | Overlaps with B3.3; consider merging the two. Including patients as active participants in monitoring may enable more timely and proactive risk detection. | Modified and merged with B3.3; represented as round-2 B2.1 | B2.1 Daily lower-limb monitoring: While nursing staff perform assessments, patients should also be educated and encouraged to act as the first reporters of skin-safety problems. Patients should be taught to recognize warning signs such as local pain, numbness, color changes, or persistent pressure marks and instructed to report them immediately. Nursing staff should assess promptly after a report and, when necessary, stop use and document and manage the problem according to protocol. |
| B2.4 | Level 3 | B2.4 Optimize positioning and early functional exercise: Elevate the lower extremities (20°–30°), avoid placing a hard pillow beneath the knees and excessive hip flexion to promote venous return; encourage patients to begin active/passive in-bed activities as early as possible, such as ankle-pump exercises and quadriceps contractions. | 4.80±0.414 (0.086) | 4.27±0.961 (0.225) | Recommended deleting “avoid placing a hard pillow under the knees and excessive hip flexion.” | Modified; renumbered as round-2 B2.2 | B2.2 Optimize positioning and early functional exercise: Promote venous return by elevating the lower limbs (20°–30°); encourage patients to begin active/passive in-bed activities as early as possible, such as ankle-pump exercises and quadriceps contractions. |
| B3 | Level 2 | Mechanical prophylaxis | 4.60±0.632 (0.137) | 4.47±0.743 (0.166) | None | Retained without change. | Unchanged from round 1. |
| B3.1 | Level 3 | B3.1 Screen for contraindications before mechanical prophylaxis: Before using graduated compression stockings (GCS) or intermittent pneumatic compression (IPC) for mechanical prophylaxis, assess for contraindications such as peripheral arterial disease and skin damage. | 4.80±0.414 (0.086) | 4.40±0.910 (0.207) | None | Retained; renumbered as round-2 B3.2 | Unchanged wording; renumbered as B3.2 in round 2. |
| B3.2 | Level 3 | B3.2 Suitability and management of mechanical prophylaxis: For patients at high risk of bleeding who are temporarily unable to receive pharmacological prophylaxis, mechanical prophylaxis alone (e.g., GCS or IPC) may be used. The regimen should be adjusted according to patient tolerance. IPC is recommended for a cumulative duration of at least 18 hours per day; for completely bedridden patients, use should be extended as much as possible to improve the preventive effect. Once bleeding risk decreases, patients should be reassessed promptly and switched to or combined with pharmacological prophylaxis. | 4.07±0.884 (0.217) | 3.53±1.187 (0.336) | The duration should be reconsidered. Although the recommendation of IPC use for at least 18 hours/day is supported by guidelines and evidence, its feasibility in routine practice may be low and may exceed many patients’ tolerance and ability to comply. | Modified; renumbered as round-2 B3.3 | B3.3 Applicability and management of mechanical prophylaxis: For patients at high bleeding risk who temporarily cannot receive pharmacological prophylaxis, mechanical prophylaxis alone (e.g., GCS or IPC) may be used. The regimen should be individualized according to patient tolerance. Use for as long as possible while the patient is awake and bedridden is recommended. In clinical practice, adequate cumulative daily use should be ensured and actual duration recorded. For completely bedridden patients, near-continuous use should be encouraged. When bleeding risk decreases, patients should be reassessed promptly and switched to or combined with pharmacological prophylaxis. |
| B3.3 | Level 3 | B3.3 Care during mechanical prophylaxis: Assess skin integrity at the application site daily. If pressure marks, skin pallor, or pain occurs, discontinue mechanical prophylaxis immediately and document and manage the event accordingly. | 4.80±0.414 (0.086) | 4.53±0.640 (0.141) | None | Merged with B2.3; represented as round-2 B2.1 | B2.1 Daily lower-limb monitoring: While nursing staff perform assessments, patients should also be educated and encouraged to act as the first reporters of skin-safety problems. Patients should be taught to recognize warning signs such as local pain, numbness, color changes, or persistent pressure marks and instructed to report them immediately. Nursing staff should assess promptly after a report and, when necessary, stop use and document and manage the problem according to protocol. |
| B4 | Level 2 | Pharmacological prophylaxis | 4.80±0.561 (0.117) | 4.67±0.724 (0.155) | None | Retained without change. | Unchanged from round 1. |
| B4.1 | Level 3 | B4.1 Pre-medication assessment and decision-making: Physicians should comprehensively assess the patient’s VTE and bleeding risks and inquire about and document previous medical history (including thrombotic events, bleeding history, and medication history). For patients at high thrombosis risk and low bleeding risk, an individualized pharmacological prophylaxis plan should be developed, and informed consent should be obtained before medication initiation regarding the purpose and importance of prevention and potential adverse effects. | 4.93±0.258 (0.052) | 4.73±0.458 (0.097) | None | Retained without change. | Unchanged from round 1. |
| B4.2 | Level 3 | B4.2 Optimization of medication selection and implementation of medical orders: For patients newly starting systemic chemotherapy who are at very high risk of thrombosis (Khorana score ≥2/Padua score ≥4), if there are no obvious high-risk bleeding factors or important drug–drug interactions, physicians should preferentially consider rivaroxaban or low-molecular-weight heparin for prophylactic anticoagulation and promptly issue the corresponding prophylaxis orders based on the assessment results. | 4.93±0.258 (0.052) | 4.80±0.561 (0.117) | Recommended merging with B4.3. Combining physician decision/execution with pharmacist review/optimization would more accurately and completely describe the full clinical process of developing a pharmacological prophylaxis regimen. | Modified and merged with B4.3; represented as round-2 B4.2 | B4.2 Development and review/optimization of the pharmacological prophylaxis regimen: For patients newly starting systemic chemotherapy and at high thrombotic risk (Khorana score ≥2/Padua score ≥4), if there are no obvious high-risk bleeding factors or important drug interactions, physicians should preferentially consider rivaroxaban or low-molecular-weight heparin, develop a prophylactic anticoagulation regimen, and promptly issue the prescription. Clinical pharmacists should complete prescription review within 24 hours of admission (including drug type, dose, and duration), propose optimization based on liver and kidney function, and collaborate with physicians to determine the final individualized regimen. |
| B4.3 | Level 3 | B4.3 Pharmacist prescription review: Clinical pharmacists should review anticoagulant prescriptions issued by physicians within 24 hours of admission (including medication type, dose, and treatment duration), optimize the anticoagulation regimen according to hepatic and renal function and other patient factors, and provide necessary recommendations for adjustment. | 4.93±0.258 (0.052) | 4.40±0.828 (0.188) | None | Merged with B4.2; represented as round-2 B4.2 | B4.2 Development and review/optimization of the pharmacological prophylaxis regimen: For patients newly starting systemic chemotherapy and at high thrombotic risk (Khorana score ≥2/Padua score ≥4), if there are no obvious high-risk bleeding factors or important drug interactions, physicians should preferentially consider rivaroxaban or low-molecular-weight heparin, develop a prophylactic anticoagulation regimen, and promptly issue the prescription. Clinical pharmacists should complete prescription review within 24 hours of admission (including drug type, dose, and duration), propose optimization based on liver and kidney function, and collaborate with physicians to determine the final individualized regimen. |
| B4.4 | Level 3 | B4.4 Nursing standards for medication administration: Nurses should strictly follow standardized procedures when injecting anticoagulants. Subcutaneous abdominal injection should be preferred for low-molecular-weight heparin, and prefilled low-molecular-weight heparin syringes should be used. Air should not be expelled before injection; the needle should be inserted slowly and vertically, and the injection site should not be compressed after needle withdrawal, to reduce bleeding risk. | 4.80±0.414 (0.086) | 4.47±0.743 (0.166) | None | Retained; renumbered as round-2 B4.3 | Unchanged wording; renumbered as B4.3 in round 2. |
| B5 | Level 2 | Health education and follow-up | 4.60±0.632 (0.137) | 4.13±0.915 (0.222) | None | Retained without change. | Unchanged from round 1. |
| B5.1 | Level 3 | B5.1 Admission education: On the day of admission, healthcare professionals should provide bedside health education, introduce VTE-related signs and symptoms, emphasize the importance of VTE prevention, and explain possible adverse effects of anticoagulant prophylaxis to improve patient adherence to prevention. | 4.80±0.414 (0.086) | 4.53±0.743 (0.164) | Recommended revising admission education so that clinicians first ask about VTE-related symptoms and signs and, for patients assessed as high risk, emphasize the importance of VTE prevention and explain potential adverse effects of anticoagulant prophylaxis. | Modified; retained as B5.1 | B5.1 Admission education: On the day of admission, healthcare professionals should provide bedside health education, first asking whether the patient has VTE-related symptoms and signs. For patients assessed as high risk, emphasize the importance of VTE prevention and explain the potential adverse effects of anticoagulant prophylaxis to improve prevention awareness and adherence. |
| B5.2 | Level 3 | B5.2 Guidance on compression stockings: Instruct patients on the correct selection and use of graduated compression stockings for prevention, and emphasize the importance of continuous use and precautions (e.g., putting them on and taking them off at scheduled times each day and maintaining limb cleanliness). | 4.60±0.632 (0.137) | 4.53±0.640 (0.141) | Suggested moving “compression stocking guidance” into B3 Mechanical prophylaxis for better clinical logic, and specifying that the guidance applies to patients assessed as requiring mechanical prophylaxis. | Modified and moved to Mechanical prophylaxis; represented as round-2 B3.1 | B3.1 Compression stocking guidance: For patients assessed as requiring mechanical prophylaxis, provide guidance on correct selection and use of graduated compression stockings, and emphasize the importance and precautions of continued use (e.g., regular daily application/removal and keeping the limbs clean). |
| B5.3 | Level 3 | B5.3 Dietary guidance: Provide appropriate dietary recommendations for hospitalization and after discharge, and instruct patients to maintain a low-salt, low-fat diet with balanced nutrition to reduce thrombosis risk. | 4.67±0.617 (0.132) | 4.33±0.976 (0.225) | None | Merged with B2.2; represented as round-2 B5.2 | B5.2 Dietary guidance: Provide reasonable dietary advice during hospitalization and after discharge; instruct patients to maintain a low-salt, low-fat diet and balanced nutrition (e.g., daily salt intake <5 g, cooking oil 25–30 g, added sugar <25 g, and adequate high-quality protein such as fish, poultry, eggs, milk, and soy products) to reduce thrombosis risk. |
| B5.4 | Level 3 | B5.4 Discharge guidance: At discharge, clinical pharmacists should provide specific guidance on the correct use of anticoagulants, including dose, frequency, treatment duration, precautions, and possible adverse reactions, to ensure safe and standardized medication use after discharge. | 4.87±0.352 (0.072) | 4.60±0.632 (0.137) | None | Retained; renumbered as round-2 B5.3 | Unchanged wording; renumbered as B5.3 in round 2. |
| B5.5 | Level 3 | B5.5 Establish a pharmaceutical care and follow-up system: Create individualized follow-up records and plans for patients receiving anticoagulant prophylaxis. Pharmacists should provide pharmaceutical care through regular telephone or outpatient follow-up, with particular attention to bleeding and other adverse reactions (e.g., examination of skin and mucosa, gums, conjunctiva, ecchymoses, and petechiae), changes in platelet count, and medication adherence; follow-up test results, VTE recurrence, and other long-term complications should also be documented to comprehensively evaluate preventive effectiveness and provide timely intervention and management. | 4.87±0.352 (0.072) | 4.33±0.900 (0.208) | Recommended optimizing the follow-up model by adding patient self-recording to strengthen participation in self-management. | Modified; renumbered as round-2 B5.4 | B5.4 Establish a pharmaceutical care and follow-up system: Create an individualized follow-up record and plan for patients receiving anticoagulant prophylaxis. Encourage and instruct patients to participate in self-monitoring records (e.g., medication diaries and adverse-reaction logs). Pharmacists should integrate patient records with regular telephone, outpatient, or online follow-up, focusing on bleeding and other adverse reactions (e.g., skin/mucosal or gingival bleeding and ecchymosis), changes in platelet counts, and medication adherence; follow-up test results, VTE recurrence, and other long-term complications should also be recorded to comprehensively assess prevention effectiveness and enable timely intervention. |
| C | Level 1 | Outcome indicators | 4.80±0.414 (0.086) | 4.80±0.414 (0.086) | None | Retained without change. | Unchanged from round 1. |
| C1 | Level 2 | Assessment quality indicators | 4.87±0.352 (0.072) | 4.87±0.352 (0.072) | None | Retained without change. | Unchanged from round 1. |
| C1.1 | Level 3 | C1.1 VTE risk assessment rate (proportion of hospitalized patients who undergo VTE risk assessment). | 4.93±0.258 (0.052) | 4.87±0.352 (0.072) | Recommended specifying the denominator population as hospitalized patients with lung cancer. | Modified; retained as C1.1 | C1.1 VTE risk assessment rate (proportion of hospitalized patients with lung cancer who receive VTE risk assessment). |
| C1.2 | Level 3 | C1.2 Bleeding risk assessment rate (proportion of hospitalized patients who undergo bleeding risk assessment). | 4.93±0.258 (0.052) | 4.87±0.352 (0.072) | Recommended specifying the denominator population as hospitalized patients with lung cancer. | Modified; retained as C1.2 | C1.2 Bleeding risk assessment rate (proportion of hospitalized patients with lung cancer who receive bleeding risk assessment). |
| C2 | Level 2 | Prevention quality indicators | 4.87±0.352 (0.072) | 4.87±0.352 (0.072) | None | Retained without change. | Unchanged from round 1. |
| C2.1 | Level 3 | C2.1 Pharmacological prophylaxis implementation rate (proportion of patients with indications for pharmacological prophylaxis who actually receive anticoagulant prophylaxis). | 4.80±0.414 (0.086) | 4.80±0.414 (0.086) | None | Retained without change. | Unchanged from round 1. |
| C2.2 | Level 3 | C2.2 Mechanical prophylaxis implementation rate (proportion of patients with indications for mechanical prophylaxis who actually receive mechanical prophylaxis). | 4.87±0.352 (0.072) | 4.73±0.458 (0.097) | None | Retained without change. | Unchanged from round 1. |
| C2.3 | Level 3 | C2.3 Combined prophylaxis implementation rate (proportion of patients with indications for combined prophylaxis who receive both pharmacological and mechanical prophylaxis). | 4.73±0.458 (0.097) | 4.47±0.743 (0.166) | None | Retained without change. | Unchanged from round 1. |
| C3 | Level 2 | Outcome quality indicators | 4.93±0.258 (0.052) | 4.93±0.258 (0.052) | None | Retained without change. | Unchanged from round 1. |
| C3.1 | Level 3 | C3.1 In-hospital VTE incidence (proportion of patients with lung cancer who experience a venous thromboembolic event during hospitalization). | 4.87±0.352 (0.072) | 4.60±0.632 (0.137) | None | Retained without change. | Unchanged from round 1. |
| C3.2 | Level 3 | C3.2 Incidence of bleeding events (proportion of patients who experience bleeding adverse events during prevention, including minor and major bleeding). | 4.67±0.816 (0.175) | 4.53±0.915 (0.202) | None | Retained without change. | Unchanged from round 1. |
| C3.3 | Level 3 | C3.3 Hospital-associated VTE mortality (proportion of patients who die due to hospital-acquired VTE). | 4.80±0.414 (0.086) | 4.67±0.617 (0.132) | None | Retained without change. | Unchanged from round 1. |

**Supplementary Questionnaire S1. Original Delphi expert consultation questionnaire (Round 1)**

**Instructions**

The questionnaire comprises three consultation tables: Table 1 for level-1 indicators, Table 2 for level-2 indicators, and Table 3 for level-3 indicators. Please rate each indicator based on your professional experience and knowledge.

Importance: 5 = very important; 4 = relatively important; 3 = moderately important; 2 = relatively unimportant; 1 = very unimportant. Feasibility: 5 = very feasible; 4 = feasible; 3 = moderately feasible; 2 = infeasible; 1 = very infeasible.

If an indicator is inaccurately described or should be deleted, please provide the proposed revision or indicate “delete” in the revision/deletion comments field. If additional indicators are needed, please enter them in the space for suggested additions.

**Table 1. Level-1 indicator consultation form**

| **Indicator** | **Importance score 5 4 3 2 1** | **Feasibility score 5 4 3 2 1** | **Revision/deletion comments** |
| --- | --- | --- | --- |
| A. Structure indicators |  |  |  |
| B. Process indicators |  |  |  |
| C. Outcome indicators |  |  |  |
| Suggested additions |  |  |  |

**Table 2. Level-2 indicator consultation form**

| **Level-1 indicator** | **Level-2 indicator** | **Importance score 5 4 3 2 1** | **Feasibility score 5 4 3 2 1** | **Revision/deletion comments** |
| --- | --- | --- | --- | --- |
| A. Structure indicators | A1. Policies and procedures |  |  |  |
| A. Structure indicators | A2. Human resources |  |  |  |
| A. Structure indicators | A3. Material resources |  |  |  |
| A. Structure indicators | Suggested additions |  |  |  |
| B. Process indicators | B1. Risk assessment and stratification |  |  |  |
| B. Process indicators | B2. Basic prevention |  |  |  |
| B. Process indicators | B3. Mechanical prophylaxis |  |  |  |
| B. Process indicators | B4. Pharmacological prophylaxis |  |  |  |
| B. Process indicators | B5. Health education and follow-up |  |  |  |
| B. Process indicators | Suggested additions |  |  |  |
| C. Outcome indicators | C1. Assessment quality indicators |  |  |  |
| C. Outcome indicators | C2. Prevention quality indicators |  |  |  |
| C. Outcome indicators | C3. Outcome quality indicators |  |  |  |
| C. Outcome indicators | Suggested additions |  |  |  |

**Table 3. Level-3 indicator consultation form**

| **Level-1 indicator** | **Level-2 indicator** | **Level-3 indicator** | **Importance score 5 4 3 2 1** | **Feasibility score 5 4 3 2 1** | **Revision/deletion comments** |
| --- | --- | --- | --- | --- | --- |
| A. Structure indicators | A1. Policies and procedures | A1.1 Develop and implement, through team collaboration, a standardized VTE prevention management process covering the entire treatment course of patients with lung cancer. The process should include specifications for risk assessment, prevention decision-making, medication implementation and monitoring, patient education, and emergency management. The team leader should supervise process implementation, develop improvement plans, and carry out continuous quality improvement. |  |  |  |
| A. Structure indicators | A1. Policies and procedures | A1.2 Incorporate VTE prevention management into the hospital quality management system and performance assessment indicators. Establish unified policies for the prevention and management of lung cancer-associated VTE, regularly evaluate the implementation of preventive measures in relevant departments, and provide timely feedback and improvement. |  |  |  |
| A. Structure indicators | A2. Human resources | A2.1 Establish a multidisciplinary VTE prevention team. Core members should include physicians from respiratory and critical care medicine/medical oncology, vascular surgeons (as appropriate), clinical pharmacists, and nurses from respiratory medicine/medical oncology. |  |  |  |
| A. Structure indicators | A2. Human resources | A2.2 Define the specific responsibilities of team members: (1) Physicians should play the central management role, be responsible for comprehensive patient assessment, assess VTE risk using tools such as the Khorana score/Padua score, integrate team recommendations, and formulate prevention plans according to patient factors; (2) Pharmacists should review the appropriateness of anticoagulant prophylaxis and optimize medication safety, with particular attention to bleeding tendency and drug–drug interactions, review individualized doses (especially for patients with hepatic or renal impairment), monitor adverse reactions, and participate in medication education and follow-up for anticoagulants; (3) Nurses should assist physicians with VTE risk assessment, implement preventive measures, provide basic prevention education, deliver individualized health education to patients and their families, and standardize documentation of the implementation of preventive measures and patient responses. |  |  |  |
| A. Structure indicators | A2. Human resources | A2.3 Provide diversified training to improve healthcare professionals’ ability to screen and assess VTE risk, manage prevention, and provide health education. Regularly assess healthcare professionals’ knowledge and skills related to VTE prevention and continuously reinforce training according to the assessment results. |  |  |  |
| A. Structure indicators | A3. Material resources | A3.1 Establish an electronic VTE risk assessment system (integrating thrombosis risk assessment tools such as the Khorana score/Padua score and bleeding risk assessment tools such as the IMPROVE score), and embed risk assessment into the electronic medical record to prompt healthcare professionals to intervene in a timely manner. |  |  |  |
| A. Structure indicators | A3. Material resources | A3.2 Ensure an adequate supply of anticoagulants required for VTE prevention (e.g., low-molecular-weight heparin and rivaroxaban) to ensure medication accessibility. |  |  |  |
| A. Structure indicators | A3. Material resources | A3.3 Provide necessary mechanical prophylaxis equipment (graduated compression stockings and intermittent pneumatic compression devices) to meet patients’ needs for mechanical prophylaxis. |  |  |  |
| A. Structure indicators | A3. Material resources | A3.4 Educational materials: Provide patients with educational booklets on VTE prevention and management, and prepare corresponding video materials for patients and their families to ensure that they understand basic prevention knowledge. |  |  |  |
| A. Structure indicators | Suggested additions |  |  |  |  |
| B. Process indicators | B1. Risk assessment and stratification | B1.1 Assess the risk of thrombosis and bleeding within 24 hours of admission. If changes in the patient’s condition may alter the thrombosis risk level, reassessment should be performed promptly. |  |  |  |
| B. Process indicators | B1. Risk assessment and stratification | B1.2 Assessment tools: The Khorana score/Padua score is recommended for assessing thrombosis risk, and the IMPROVE bleeding risk score is recommended for assessing bleeding risk. |  |  |  |
| B. Process indicators | B1. Risk assessment and stratification | B1.3 For patients with suspected lower-extremity deep vein thrombosis (DVT), lower-extremity venous ultrasonography is the preferred imaging screening method. |  |  |  |
| B. Process indicators | B1. Risk assessment and stratification | B1.4 D-dimer testing may assist in screening for acute pulmonary thromboembolism (PTE). When D-dimer is markedly elevated, pulmonary artery CT angiography or lower-extremity venous ultrasonography should be completed within 12 hours. |  |  |  |
| B. Process indicators | B1. Risk assessment and stratification | B1.5 For hospitalized patients assessed as high risk, place a “thrombosis prevention warning sign” at the bedside and verify the implementation of preventive measures during the daily morning handover. |  |  |  |
| B. Process indicators | B2. Basic prevention | B2.1 Fluid intake: When clinically appropriate, ensure an adequate daily fluid intake (approximately 1500–2500 mL of water; adjust as appropriate for patients with cardiac insufficiency). |  |  |  |
| B. Process indicators | B2. Basic prevention | B2.2 Lifestyle modification: Provide guidance and support for smoking and alcohol cessation, assist patients in developing healthy lifestyle habits, formulate a low-salt, low-sugar, low-fat, high-protein diet plan, and supervise its implementation. |  |  |  |
| B. Process indicators | B2. Basic prevention | B2.3 Monitor the lower extremities daily: Assess both lower extremities for swelling, changes in skin temperature, abnormal coloration, pain, and other abnormalities. If abnormalities are identified, report them to the healthcare team within 2 hours and manage them promptly. |  |  |  |
| B. Process indicators | B2. Basic prevention | B2.4 Optimize positioning and early functional exercise: Elevate the lower extremities (20°–30°), avoid placing a hard pillow beneath the knees and excessive hip flexion to promote venous return; encourage patients to begin active/passive in-bed activities as early as possible, such as ankle-pump exercises and quadriceps contractions. |  |  |  |
| B. Process indicators | B3. Mechanical prophylaxis | B3.1 Screen for contraindications before mechanical prophylaxis: Before using graduated compression stockings (GCS) or intermittent pneumatic compression (IPC) for mechanical prophylaxis, assess for contraindications such as peripheral arterial disease and skin damage. |  |  |  |
| B. Process indicators | B3. Mechanical prophylaxis | B3.2 Suitability and management of mechanical prophylaxis: For patients at high risk of bleeding who are temporarily unable to receive pharmacological prophylaxis, mechanical prophylaxis alone (e.g., GCS or IPC) may be used. The regimen should be adjusted according to patient tolerance. IPC is recommended for a cumulative duration of at least 18 hours per day; for completely bedridden patients, use should be extended as much as possible to improve the preventive effect. Once bleeding risk decreases, patients should be reassessed promptly and switched to or combined with pharmacological prophylaxis. |  |  |  |
| B. Process indicators | B3. Mechanical prophylaxis | B3.3 Care during mechanical prophylaxis: Assess skin integrity at the application site daily. If pressure marks, skin pallor, or pain occurs, discontinue mechanical prophylaxis immediately and document and manage the event accordingly. |  |  |  |
| B. Process indicators | B4. Pharmacological prophylaxis | B4.1 Pre-medication assessment and decision-making: Physicians should comprehensively assess the patient’s VTE and bleeding risks and inquire about and document previous medical history (including thrombotic events, bleeding history, and medication history). For patients at high thrombosis risk and low bleeding risk, an individualized pharmacological prophylaxis plan should be developed, and informed consent should be obtained before medication initiation regarding the purpose and importance of prevention and potential adverse effects. |  |  |  |
| B. Process indicators | B4. Pharmacological prophylaxis | B4.2 Optimization of medication selection and implementation of medical orders: For patients newly starting systemic chemotherapy who are at very high risk of thrombosis (Khorana score ≥2/Padua score ≥4), if there are no obvious high-risk bleeding factors or important drug–drug interactions, physicians should preferentially consider rivaroxaban or low-molecular-weight heparin for prophylactic anticoagulation and promptly issue the corresponding prophylaxis orders based on the assessment results. |  |  |  |
| B. Process indicators | B4. Pharmacological prophylaxis | B4.3 Pharmacist prescription review: Clinical pharmacists should review anticoagulant prescriptions issued by physicians within 24 hours of admission (including medication type, dose, and treatment duration), optimize the anticoagulation regimen according to hepatic and renal function and other patient factors, and provide necessary recommendations for adjustment. |  |  |  |
| B. Process indicators | B4. Pharmacological prophylaxis | B4.4 Nursing standards for medication administration: Nurses should strictly follow standardized procedures when injecting anticoagulants. Subcutaneous abdominal injection should be preferred for low-molecular-weight heparin, and prefilled low-molecular-weight heparin syringes should be used. Air should not be expelled before injection; the needle should be inserted slowly and vertically, and the injection site should not be compressed after needle withdrawal, to reduce bleeding risk. |  |  |  |
| B. Process indicators | B5. Health education and follow-up | B5.1 Admission education: On the day of admission, healthcare professionals should provide bedside health education, introduce VTE-related signs and symptoms, emphasize the importance of VTE prevention, and explain possible adverse effects of anticoagulant prophylaxis to improve patient adherence to prevention. |  |  |  |
| B. Process indicators | B5. Health education and follow-up | B5.2 Guidance on compression stockings: Instruct patients on the correct selection and use of graduated compression stockings for prevention, and emphasize the importance of continuous use and precautions (e.g., putting them on and taking them off at scheduled times each day and maintaining limb cleanliness). |  |  |  |
| B. Process indicators | B5. Health education and follow-up | B5.3 Dietary guidance: Provide appropriate dietary recommendations for hospitalization and after discharge, and instruct patients to maintain a low-salt, low-fat diet with balanced nutrition to reduce thrombosis risk. |  |  |  |
| B. Process indicators | B5. Health education and follow-up | B5.4 Discharge guidance: At discharge, clinical pharmacists should provide specific guidance on the correct use of anticoagulants, including dose, frequency, treatment duration, precautions, and possible adverse reactions, to ensure safe and standardized medication use after discharge. |  |  |  |
| B. Process indicators | B5. Health education and follow-up | B5.5 Establish a pharmaceutical care and follow-up system: Create individualized follow-up records and plans for patients receiving anticoagulant prophylaxis. Pharmacists should provide pharmaceutical care through regular telephone or outpatient follow-up, with particular attention to bleeding and other adverse reactions (e.g., examination of skin and mucosa, gums, conjunctiva, ecchymoses, and petechiae), changes in platelet count, and medication adherence; follow-up test results, VTE recurrence, and other long-term complications should also be documented to comprehensively evaluate preventive effectiveness and provide timely intervention and management. |  |  |  |
| B. Process indicators | Suggested additions |  |  |  |  |
| C. Outcome indicators | C1. Assessment quality indicators | C1.1 VTE risk assessment rate (proportion of hospitalized patients who undergo VTE risk assessment). |  |  |  |
| C. Outcome indicators | C1. Assessment quality indicators | C1.2 Bleeding risk assessment rate (proportion of hospitalized patients who undergo bleeding risk assessment). |  |  |  |
| C. Outcome indicators | C2. Prevention quality indicators | C2.1 Pharmacological prophylaxis implementation rate (proportion of patients with indications for pharmacological prophylaxis who actually receive anticoagulant prophylaxis). |  |  |  |
| C. Outcome indicators | C2. Prevention quality indicators | C2.2 Mechanical prophylaxis implementation rate (proportion of patients with indications for mechanical prophylaxis who actually receive mechanical prophylaxis). |  |  |  |
| C. Outcome indicators | C2. Prevention quality indicators | C2.3 Combined prophylaxis implementation rate (proportion of patients with indications for combined prophylaxis who receive both pharmacological and mechanical prophylaxis). |  |  |  |
| C. Outcome indicators | C3. Outcome quality indicators | C3.1 In-hospital VTE incidence (proportion of patients with lung cancer who experience a venous thromboembolic event during hospitalization). |  |  |  |
| C. Outcome indicators | C3. Outcome quality indicators | C3.2 Incidence of bleeding events (proportion of patients who experience bleeding adverse events during prevention, including minor and major bleeding). |  |  |  |
| C. Outcome indicators | C3. Outcome quality indicators | C3.3 Hospital-associated VTE mortality (proportion of patients who die due to hospital-acquired VTE). |  |  |  |
| C. Outcome indicators | Suggested additions |  |  |  |  |

**Expert background information**

| **Name / Sex / Age** | ____________________________________________ |
| --- | --- |
| **Education** | □ Doctorate □ Master’s □ Bachelor’s □ Other: __________ |
| **Professional title** | □ Senior □ Associate senior □ Intermediate □ Other: __________ |
| **Years of professional experience** | __________ |
| **Professional field** | □ Clinical pharmacy □ Medical oncology □ Respiratory medicine □ Vascular surgery □ Nursing management □ Pharmacy education □ Other: __________ |
| **Contact information (email)** | ____________________________________________ |
|  |  |

**Basis for expert judgment**

| **Basis for judgment** | **High influence** | **Moderate influence** | **Low influence** |
| --- | --- | --- | --- |
| Theoretical analysis | □ | □ | □ |
| Practical experience | □ | □ | □ |
| Domestic and international literature | □ | □ | □ |
| Intuition | □ | □ | □ |

**Familiarity with the questionnaire content**

|  | **Very familiar** | **Familiar** | **Moderately familiar** | **Not very familiar** | **Not familiar** |
| --- | --- | --- | --- | --- | --- |
| Self-assessment | □ | □ | □ | □ | □ |

**Supplementary Questionnaire S2. Original Delphi expert consultation questionnaire (Round 2)**

**Instructions**

The questionnaire comprises three consultation tables: Table 1 for level-1 indicators, Table 2 for level-2 indicators, and Table 3 for level-3 indicators. Please rate each indicator based on your professional experience and knowledge.

Importance: 5 = very important; 4 = relatively important; 3 = moderately important; 2 = relatively unimportant; 1 = very unimportant. Feasibility: 5 = very feasible; 4 = feasible; 3 = moderately feasible; 2 = infeasible; 1 = very infeasible.

If an indicator is inaccurately described or should be deleted, please provide the proposed revision or indicate “delete” in the revision/deletion comments field. If additional indicators are needed, please enter them in the space for suggested additions.

**Table 1. Level-1 indicator consultation form**

| **Indicator** | **Importance score 5 4 3 2 1** | **Feasibility score 5 4 3 2 1** | **Revision/deletion comments** |
| --- | --- | --- | --- |
| A. Structure indicators |  |  |  |
| B. Process indicators |  |  |  |
| C. Outcome indicators |  |  |  |
| Suggested additions |  |  |  |

**Table 2. Level-2 indicator consultation form**

| **Level-1 indicator** | **Level-2 indicator** | **Importance score 5 4 3 2 1** | **Feasibility score 5 4 3 2 1** | **Revision/deletion comments** |
| --- | --- | --- | --- | --- |
| A. Structure indicators | A1. Policies and procedures |  |  |  |
| A. Structure indicators | A2. Human resources |  |  |  |
| A. Structure indicators | A3. Material resources |  |  |  |
| A. Structure indicators | Suggested additions |  |  |  |
| B. Process indicators | B1. Risk assessment and stratification |  |  |  |
| B. Process indicators | B2. Basic prevention |  |  |  |
| B. Process indicators | B3. Mechanical prophylaxis |  |  |  |
| B. Process indicators | B4. Pharmacological prophylaxis |  |  |  |
| B. Process indicators | B5. Health education and follow-up |  |  |  |
| B. Process indicators | Suggested additions |  |  |  |
| C. Outcome indicators | C1. Assessment quality indicators |  |  |  |
| C. Outcome indicators | C2. Prevention quality indicators |  |  |  |
| C. Outcome indicators | C3. Outcome quality indicators |  |  |  |
| C. Outcome indicators | Suggested additions |  |  |  |

**Table 3. Level-3 indicator consultation form**

| **Level-1 indicator** | **Level-2 indicator** | **Level-3 indicator** | **Importance score 5 4 3 2 1** | **Feasibility score 5 4 3 2 1** | **Revision/deletion comments** |
| --- | --- | --- | --- | --- | --- |
| A. Structure indicators | A1. Policies and procedures | A1.1 Develop and implement, through team collaboration, a standardized VTE prevention management process covering the entire treatment course of patients with lung cancer. The process should include specifications for risk assessment, prevention decision-making, medication implementation and monitoring, patient education, and emergency management. The team leader should supervise process implementation, develop improvement plans, and carry out continuous quality improvement. |  |  |  |
| A. Structure indicators | A1. Policies and procedures | A1.2 Incorporate VTE prevention management into the hospital quality management system and performance assessment indicators. Establish unified policies for the prevention and management of lung cancer-associated VTE, regularly evaluate the implementation of preventive measures in relevant departments, and provide timely feedback and improvement. |  |  |  |
| A. Structure indicators | A2. Human resources | A2.1 Establish a multidisciplinary VTE prevention team. Core members should include physicians from respiratory and critical care medicine/medical oncology, vascular surgeons (as appropriate), clinical pharmacists, and nurses from respiratory medicine/medical oncology. |  |  |  |
| A. Structure indicators | A2. Human resources | A2.2 Define the specific responsibilities of team members: (1) Physicians should play the central management role, be responsible for comprehensive patient assessment, assess VTE risk using tools such as the Khorana score/Padua score, integrate team recommendations, and formulate prevention plans according to patient factors; (2) Pharmacists should review the appropriateness of anticoagulant prophylaxis and optimize medication safety, with particular attention to bleeding tendency and drug–drug interactions, review individualized doses (especially for patients with hepatic or renal impairment), monitor adverse reactions, and participate in medication education and follow-up for anticoagulants; (3) Nurses should assist physicians with VTE risk assessment, implement preventive measures, provide basic prevention education, deliver individualized health education to patients and their families, and standardize documentation of the implementation of preventive measures and patient responses. |  |  |  |
| A. Structure indicators | A2. Human resources | A2.3 Provide diversified training to improve healthcare professionals’ ability to screen and assess VTE risk, manage prevention, and provide health education. Regularly assess healthcare professionals’ knowledge and skills related to VTE prevention and continuously reinforce training according to the assessment results. |  |  |  |
| A. Structure indicators | A3. Material resources | A3.1 Establish an electronic VTE risk assessment system (integrating thrombosis risk assessment tools such as the Khorana score/Padua score and bleeding risk assessment tools such as the IMPROVE score), and embed risk assessment into the electronic medical record to prompt healthcare professionals to intervene in a timely manner. |  |  |  |
| A. Structure indicators | A3. Material resources | A3.2 Ensure an adequate supply of anticoagulants required for VTE prevention (e.g., low-molecular-weight heparin and rivaroxaban) to ensure medication accessibility. |  |  |  |
| A. Structure indicators | A3. Material resources | A3.3 Provide necessary mechanical prophylaxis equipment (graduated compression stockings and intermittent pneumatic compression devices) to meet patients’ needs for mechanical prophylaxis. |  |  |  |
| A. Structure indicators | A3. Material resources | A3.4 Educational materials: Provide patients with educational booklets on VTE prevention and management, and prepare corresponding video materials for patients and their families to ensure that they understand basic prevention knowledge. |  |  |  |
| A. Structure indicators | Suggested additions |  |  |  |  |
| B. Process indicators | B1. Risk assessment and stratification | B1.1 Assess the risk of thrombosis and bleeding within 24 hours of admission. If changes in the patient’s condition may alter the thrombosis risk level, reassessment should be performed promptly. |  |  |  |
| B. Process indicators | B1. Risk assessment and stratification | B1.2 Assessment tools: The Khorana score/Padua score is recommended for assessing thrombosis risk, and the IMPROVE bleeding risk score is recommended for assessing bleeding risk. |  |  |  |
| B. Process indicators | B1. Risk assessment and stratification | B1.3 For patients with suspected lower-extremity deep vein thrombosis (DVT), lower-extremity venous ultrasonography is the preferred imaging screening method. |  |  |  |
| B. Process indicators | B1. Risk assessment and stratification | B1.4 D-dimer testing may assist in screening for acute pulmonary thromboembolism (PTE). When D-dimer is markedly elevated, urgent management should be undertaken according to the clinical situation: for patients with high clinical suspicion of PTE, an emergency diagnostic pathway should be initiated and pulmonary artery CT angiography actively coordinated within 12 hours; for those with low-to-moderate suspicion, lower-extremity venous ultrasonography and other examinations should be arranged as soon as possible, with close monitoring for changes in condition. |  |  |  |
| B. Process indicators | B1. Risk assessment and stratification | B1.5 For hospitalized patients receiving thrombosis prevention and treatment, place a “thrombosis prevention warning sign” at the bedside and verify implementation of preventive measures during the daily morning handover. |  |  |  |
| B. Process indicators | B2. Basic prevention | B2.1 Daily lower-limb monitoring: While nursing staff perform assessments, patients should also be educated and encouraged to act as the first reporters of skin-safety problems. Patients should be taught to recognize warning signs such as local pain, numbness, color changes, or persistent pressure marks and instructed to report them immediately. Nursing staff should assess promptly after a report and, when necessary, stop use and document and manage the problem according to protocol. |  |  |  |
| B. Process indicators | B2. Basic prevention | B2.2 Optimize positioning and early functional exercise: Promote venous return by elevating the lower limbs (20°–30°); encourage patients to begin active/passive in-bed activities as early as possible, such as ankle-pump exercises and quadriceps contractions. |  |  |  |
| B. Process indicators | B3. Mechanical prophylaxis | B3.1 Compression stocking guidance: For patients assessed as requiring mechanical prophylaxis, provide guidance on correct selection and use of graduated compression stockings, and emphasize the importance and precautions of continued use (e.g., regular daily application/removal and keeping the limbs clean). |  |  |  |
| B. Process indicators | B3. Mechanical prophylaxis | B3.2 Screen for contraindications before mechanical prophylaxis: Before using graduated compression stockings (GCS) or intermittent pneumatic compression (IPC) for mechanical prophylaxis, assess for contraindications such as peripheral arterial disease and skin damage. |  |  |  |
| B. Process indicators | B3. Mechanical prophylaxis | B3.3 Applicability and use management of mechanical prophylaxis: For patients at high bleeding risk who temporarily cannot receive pharmacological prophylaxis, mechanical prophylaxis alone (e.g., GCS or IPC) may be used. The regimen should be individualized according to patient tolerance. Mechanical prophylaxis should be used for as long as possible while the patient is awake and in bed. In clinical practice, sufficient cumulative daily use should be ensured and actual duration documented. For patients who are completely bedridden, near-continuous use should be encouraged. Once bleeding risk decreases, patients should be reassessed promptly and switched to or combined with pharmacological prophylaxis. |  |  |  |
| B. Process indicators | B4. Pharmacological prophylaxis | B4.1 Pre-medication assessment and decision-making: Physicians should comprehensively assess the patient’s VTE and bleeding risks and inquire about and document previous medical history (including thrombotic events, bleeding history, and medication history). For patients at high thrombosis risk and low bleeding risk, an individualized pharmacological prophylaxis plan should be developed, and informed consent should be obtained before medication initiation regarding the purpose and importance of prevention and potential adverse effects. |  |  |  |
| B. Process indicators | B4. Pharmacological prophylaxis | B4.2 Development and review/optimization of the pharmacological prophylaxis regimen: For patients newly starting systemic chemotherapy and at high thrombotic risk (Khorana score ≥2/Padua score ≥4), if there are no obvious high-risk bleeding factors or important drug interactions, physicians should preferentially consider rivaroxaban or low-molecular-weight heparin, develop a prophylactic anticoagulation regimen, and promptly issue the prescription. Clinical pharmacists should complete prescription review within 24 hours of admission (including drug type, dose, and duration), propose optimization based on liver and kidney function, and collaborate with physicians to determine the final individualized regimen. |  |  |  |
| B. Process indicators | B4. Pharmacological prophylaxis | B4.3 Nursing standards for medication administration: Nurses should strictly follow standardized procedures when injecting anticoagulants. Subcutaneous abdominal injection should be preferred for low-molecular-weight heparin, and prefilled low-molecular-weight heparin syringes should be used. Air should not be expelled before injection; the needle should be inserted slowly and vertically, and the injection site should not be compressed after needle withdrawal, to reduce bleeding risk. |  |  |  |
| B. Process indicators | B5. Health education and follow-up | B5.1 Admission education: On the day of admission, healthcare professionals should provide bedside health education, first asking whether the patient has VTE-related symptoms and signs. For patients assessed as high risk, emphasize the importance of VTE prevention and explain the potential adverse effects of anticoagulant prophylaxis to improve prevention awareness and adherence. |  |  |  |
| B. Process indicators | B5. Health education and follow-up | B5.2 Dietary guidance: Provide reasonable dietary advice during hospitalization and after discharge; instruct patients to maintain a low-salt, low-fat diet and balanced nutrition (e.g., daily salt intake <5 g, cooking oil 25–30 g, added sugar intake <25 g, and adequate high-quality protein such as fish, poultry, eggs, milk, and soy products) to reduce thrombosis risk. |  |  |  |
| B. Process indicators | B5. Health education and follow-up | B5.3 Discharge guidance: At discharge, clinical pharmacists should provide specific guidance on the correct use of anticoagulants, including dose, frequency, treatment duration, precautions, and possible adverse reactions, to ensure safe and standardized medication use after discharge. |  |  |  |
| B. Process indicators | B5. Health education and follow-up | B5.4 Establish a pharmaceutical care and follow-up system: Create an individualized follow-up record and plan for patients receiving anticoagulant prophylaxis. Encourage and instruct patients to participate in self-monitoring records (e.g., medication diaries and adverse-reaction logs). Pharmacists should integrate patient records with regular telephone, outpatient, or online follow-up, focusing on bleeding and other adverse reactions (e.g., skin/mucosal or gingival bleeding and ecchymosis), changes in platelet counts, and medication adherence; follow-up test results, VTE recurrence, and other long-term complications should also be recorded to comprehensively assess prevention effectiveness and enable timely intervention. |  |  |  |
| B. Process indicators | Suggested additions |  |  |  |  |
| C. Outcome indicators | C1. Assessment quality indicators | C1.1 VTE risk assessment rate (proportion of hospitalized patients with lung cancer who receive VTE risk assessment). |  |  |  |
| C. Outcome indicators | C1. Assessment quality indicators | C1.2 Bleeding risk assessment rate (proportion of hospitalized patients with lung cancer who receive bleeding risk assessment). |  |  |  |
| C. Outcome indicators | C2. Prevention quality indicators | C2.1 Pharmacological prophylaxis implementation rate (proportion of patients with indications for pharmacological prophylaxis who actually receive anticoagulant prophylaxis). |  |  |  |
| C. Outcome indicators | C2. Prevention quality indicators | C2.2 Mechanical prophylaxis implementation rate (proportion of patients with indications for mechanical prophylaxis who actually receive mechanical prophylaxis). |  |  |  |
| C. Outcome indicators | C2. Prevention quality indicators | C2.3 Combined prophylaxis implementation rate (proportion of patients with indications for combined prophylaxis who receive both pharmacological and mechanical prophylaxis). |  |  |  |
| C. Outcome indicators | C3. Outcome quality indicators | C3.1 In-hospital VTE incidence (proportion of patients with lung cancer who experience a venous thromboembolic event during hospitalization). |  |  |  |
| C. Outcome indicators | C3. Outcome quality indicators | C3.2 Incidence of bleeding events (proportion of patients who experience bleeding adverse events during prevention, including minor and major bleeding). |  |  |  |
| C. Outcome indicators | C3. Outcome quality indicators | C3.3 In-hospital VTE-related mortality (proportion of patients who die due to hospital-acquired VTE). |  |  |  |
| C. Outcome indicators | Suggested additions |  |  |  |  |

**Expert background information**

| **Name / Sex / Age** | ____________________________________________ |
| --- | --- |
| **Education** | □ Doctorate □ Master’s □ Bachelor’s □ Other: __________ |
| **Professional title** | □ Senior □ Associate senior □ Intermediate □ Other: __________ |
| **Years of professional experience** | __________ |
| **Professional field** | □ Clinical pharmacy □ Medical oncology □ Respiratory medicine □ Vascular surgery □ Nursing management □ Pharmacy education □ Other: __________ |
| **Contact information (email)** | ____________________________________________ |
|  |  |

**Basis for expert judgment**

| **Basis for judgment** | **High influence** | **Moderate influence** | **Low influence** |
| --- | --- | --- | --- |
| Theoretical analysis | □ | □ | □ |
| Practical experience | □ | □ | □ |
| Domestic and international literature | □ | □ | □ |
| Intuition | □ | □ | □ |

**Familiarity with the questionnaire content**

|  | **Very familiar** | **Familiar** | **Moderately familiar** | **Not very familiar** | **Not familiar** |
| --- | --- | --- | --- | --- | --- |
| Self-assessment | □ | □ | □ | □ | □ |

**Supplementary Table S4. Source-derived information and proposed operational specifications for framework measures**

*Definitions selected for manuscript revision — provisional specifications for future implementation testing; not externally validated.*

| Framework code and measure | Source-derived definition / information | Proposed calculation formula | Eligibility and exclusions | Assessment window / counting | Data source and responsibility | Appropriateness criteria / documentation | Interpretation and adjustment | Change and validation status | Frequency and governance |
| --- | --- | --- | --- | --- | --- | --- | --- | --- | --- |
| Common proposed measurement rules — Unit and formula: use admission episodes consistently. Calculate a proportion as qualifying admissions / eligible admissions × 100. Repeated admissions are separate episodes; report unique patients as contextual information. Count an admission once for each binary measure. Timing and frequency: capture assessments, delivery and events contemporaneously; verify after discharge/death and aggregate by discharge month. Missing data: distinguish not documented, confirmed not performed, unknown eligibility and unknown outcome; report missingness and analyzable denominators. Zero denominator: report not applicable, with numerator and denominator shown. Changing eligibility: record eligible intervals and changes in risk, contraindications and anticoagulation status. Implementation prerequisites: finalize a versioned clinical eligibility protocol, device/drug-specific contraindications, timing/dose criteria, event manual and data dictionary before prospective collection. Risk adjustment: no model was developed or validated; comparative use requires attention to case mix, time at risk and ascertainment. | | | | | | | | | |
| C1.1 — VTE risk assessment rate | Proportion of hospitalized patients with lung cancer who complete VTE risk assessment using the Padua score and/or Khorana score within 24 h of admission. Original denominator: total hospitalized patients with lung cancer included in management during the same period. | Proportion of eligible admissions with a completed VTE risk assessment within 24 h. Numerator: admissions with recorded assessment tool/version, completed score, risk category, and timestamp within the window. | All admissions in the framework cohort during the reporting period. Parent study excluded incomplete information preventing risk scoring; proposed quality audit retains these admissions and reports unassessable status separately. | Admission to 24 h after admission. Admissions ending before 24 h remain in the cohort and are assessed against completion before discharge; report separately. | Source: paper risk-assessment form and clinical record; clinical pharmacist assessed and physician reviewed. Proposed: link to admission/discharge timestamps and retain an auditable record. | Not separately specified for this completion measure. | Higher documented completion is desirable. Completion does not establish predictive validity. No risk adjustment proposed; report missing-data and short-stay strata. | 24-h window is source-derived. Completion fields, admission-based counting, short-stay handling and missing-data rules are proposed. Padua and/or Khorana use is historical, not a recommendation that they are interchangeable. | Proposed: capture during care, verify after discharge/death, aggregate monthly. Hospital quality team coordinates extraction/audit with clinical teams; local staff assignment must be agreed. |
| C1.2 — Bleeding risk assessment rate | Proportion of hospitalized patients with lung cancer at intermediate/high VTE risk who complete IMPROVE bleeding-risk assessment within 24 h of admission. Original denominator: total patients assessed as intermediate/high VTE risk. | Proportion of source-defined eligible admissions with completed bleeding-risk assessment within 24 h. Numerator: eligible admissions with recorded IMPROVE assessment, score, risk category, and timestamp within 24 h. | Admissions categorized as intermediate/high VTE risk under the parent-study definition. Other VTE-risk categories are outside this denominator. Unknown VTE-risk classification should be reported separately. | Within 24 h of admission. Subsequent reassessment is not counted as timely initial completion. | Source: paper IMPROVE assessment form and clinical record; clinical pharmacist assessed, physician used the result. Proposed: verify timestamps against admission time. | Not separately specified for this completion measure. | Higher timely completion in the defined denominator is desirable. No risk adjustment proposed. Report unknown eligibility and admissions discharged before the window ends. | Original denominator and 24-h window retained. Required completion fields and explicit missing-eligibility reporting are proposed. Denominator documents the parent study, not a universal eligibility rule. | Proposed: capture during care, verify after discharge/death, aggregate monthly. Hospital quality team coordinates extraction/audit; local staff assignment must be agreed. |
| C2.1 — Appropriate pharmacological prophylaxis among eligible patients | Proportion of hospitalized patients with lung cancer at intermediate/high VTE risk (e.g., Padua ≥4 or Khorana ≥2) and low bleeding risk by IMPROVE who actually receive prophylactic anticoagulation during hospitalization. Original denominator: intermediate/high VTE risk and low bleeding risk. | Proposed revised measure: proportion of eligible admissions receiving appropriate pharmacological prophylaxis under a specified guideline-based institutional protocol. Numerator requires documented actual delivery and conformity with applicable clinical criteria, including justified withholding/interruption. | Proposed: documented indication for pharmacological primary prophylaxis and at least one interval without a relevant contraindication. Exclude therapeutic anticoagulation throughout, no indication, or contraindicated throughout. Catheter-locking and other non-prophylactic uses do not count. | Index hospitalization; identify eligible intervals and changes in anticoagulation status. Treatment-dose intervals do not count as prophylactic coverage. | Source: medication-order system, dose/duration records; pharmacist assessed, physician prescribed, nurses administered/monitored. Proposed: corroborate orders with administration records and contraindications. | Record thrombosis risk, bleeding risk, contraindications, prophylaxis indication and clinical rationale. Assess agent, dose, route, timing and actual administration against a named, versioned guideline-based institutional protocol. | Higher guideline-concordant delivery within the modality-specific eligible population is desirable; higher raw use across all admissions is not inherently better care. Report unnecessary use separately. No validated risk-adjustment model supplied. | Original source definition/denominator retained for transparency. Revised appropriateness measure changes receipt-based operationalization and was not evaluated in the original Delphi consultation. External measurement validation and prospective testing required. | Proposed: capture during care, verify after discharge/death, aggregate monthly. Hospital quality team coordinates extraction/audit; local staff assignment must be agreed. |
| C2.2 — Appropriate mechanical prophylaxis among eligible patients | Proportion of hospitalized patients with lung cancer at intermediate/high VTE risk for whom pharmacist risk assessment/recommendation is followed by physician confirmation of no relevant contraindication, prescription, and nursing implementation. Original denominator: all intermediate/high-risk patients. | Proposed revised measure: proportion of eligible admissions receiving appropriate mechanical prophylaxis under the specified guideline-based institutional protocol. Numerator requires documented actual delivery and conformity with applicable clinical criteria during eligible intervals. | Proposed correction: documented indication for the selected mechanical modality and no relevant device-specific contraindication during at least one interval. Exclude no indication or contraindicated throughout. Source denominator included all intermediate/high-risk patients despite narrative requirement for no contraindications. | Index hospitalization, restricted to eligible intervals. Record changes in eligibility and reasons for non-application/interruption. | Source: physician orders and nursing implementation records; pharmacist assessed risk, physician assessed/prescribed, nurses implemented. Proposed: record device-specific contraindication assessment and application/removal times. | Record thrombosis/bleeding risk, modality-specific indication and contraindications, device selection, actual application, intended duration, interruptions and rationale. A pharmacological contraindication is not automatically a mechanical contraindication. | Higher guideline-concordant delivery within the eligible population is desirable; higher raw use is not inherently better care. Report unnecessary use separately. No validated risk-adjustment model supplied. | Original source definition/denominator retained for transparency. Revised appropriateness measure changes the original receipt-based operationalization and was not Delphi-evaluated. External validation/prospective testing required. | Proposed: capture during care, verify after discharge/death, aggregate monthly. Hospital quality team coordinates extraction/audit; local staff assignment must be agreed. |
| C2.3 — Appropriate combined prophylaxis among eligible patients | Proportion of hospitalized patients with lung cancer at high VTE risk (e.g., Padua ≥4 and Khorana ≥2), low bleeding risk by IMPROVE, and no mechanical-prophylaxis contraindication who actually receive both pharmacological and mechanical prophylaxis. Original denominator: same eligibility set. | Proposed revised measure: proportion of eligible admissions receiving appropriate combined pharmacological and mechanical prophylaxis under the specified guideline-based institutional protocol. Numerator requires documented actual delivery and conformity with applicable clinical criteria during eligible intervals. | Proposed: explicitly documented indication for combined prophylaxis, no relevant contraindication to either modality during the intended interval, and no therapeutic-dose anticoagulation during that interval. High risk by score alone is not sufficient justification here. | Index hospitalization. Specify intended combined intervals in the prescription/care plan and verify delivery of each modality within those intervals. | Source: medication and nursing records; pharmacist assessed, physician prescribed, nurses delivered. Proposed: link administration/device-use records to the documented combined-prophylaxis rationale. | Record explicit indication for combination, thrombosis/bleeding risk, contraindications to each modality and rationale. Verify both components belong to the same planned preventive course and meet drug/device/timing criteria. Sequential switching alone is not combined prophylaxis. | Higher guideline-concordant delivery within the eligible population is desirable; higher raw use is not inherently better care. No validated risk-adjustment model supplied. Combined prophylaxis is not a universal target for medical inpatients. | Original source definition/denominator retained. Revised appropriateness measure was not evaluated in the original Delphi consultation. External measurement validation and prospective testing required. | Proposed: capture during care, verify after discharge/death, aggregate monthly. Hospital quality team coordinates extraction/audit; local staff assignment must be agreed. |
| C3.1 — In-hospital VTE incidence | Proportion of patients with lung cancer who, during the current hospitalization, first develop and are definitively diagnosed with hospital-associated VTE, including DVT or pulmonary embolism. Original denominator: all hospitalized patients with lung cancer during the same period. | Proposed: eligible admissions with a first newly occurring, objectively confirmed DVT or PE during the index hospitalization and not present on admission. Appropriate diagnostic imaging or autopsy evidence is required; suspicion, risk score or elevated D-dimer alone is insufficient. | Proposed: all framework-cohort admissions without active VTE present on admission. Report indeterminate admission-VTE status separately. Prior resolved VTE is a baseline characteristic rather than an automatic exclusion. | Admission through discharge or in-hospital death. Exclude events present on admission even if diagnosed later. Count each admission once in combined DVT/PE rate; record event type and symptomatic/incidental detection separately. Post-discharge events require a distinct measure. | Proposed: link imaging/autopsy reports, clinical notes, admission assessments and discharge diagnoses. Two clinician reviewers independently ascertain event status/onset; a third adjudicates disagreement. | Objective confirmation and an event manual are required; anatomical DVT coverage, including catheter-related/upper-extremity events, must be fixed before data collection. | Lower observed incidence is desirable but does not alone prove better care. Report case mix and diagnostic ascertainment. Benchmarking requires development/validation of adjustment for cancer stage, treatment, comorbidity, time at risk and diagnostic intensity. | Source specified index-hospitalization onset/diagnosis but not proposed ascertainment procedures. Admission-status rules, confirmation, counting, denominator refinement and adjudication are proposed. | Proposed: capture during care, verify after discharge/death, aggregate monthly. Hospital quality team coordinates extraction/audit; local staff assignment must be agreed. |
| C3.2 — Bleeding event rate | Proportion of hospitalized patients with lung cancer receiving prophylactic anticoagulant treatment who experience major bleeding or clinically relevant non-major bleeding during hospitalization. Original denominator: all prophylactic-anticoagulation-treated patients. | Proposed: prophylactic-anticoagulation-exposed admissions with a new in-hospital major bleeding or clinically relevant non-major bleeding event, classified using published ISTH definitions for nonsurgical patients. Report categories separately and optionally combined. | Admissions with documented prophylactic anticoagulant administration. No actual exposure = outside denominator. Bleeding before first prophylactic administration is not counted as an incident post-exposure event; a distinct subsequent event requires adjudication. | From first prophylactic administration through discharge or in-hospital death. Count once per reported category. Record onset relative to dosing, cessation and transition to therapeutic anticoagulation; later therapeutic-exposure events should be identified separately. | Source: pharmacists monitored bleeding, without a specified outcome-ascertainment department. Proposed: clinical notes, hemoglobin, transfusion/procedure and administration records; two clinician reviewers apply ISTH criteria, third resolves disagreements. | Use the full published ISTH definitions for major bleeding and clinically relevant non-major bleeding in nonsurgical patients; temporal occurrence does not establish drug causation. | Lower incidence is desirable but must be interpreted with VTE and prophylaxis appropriateness. Comparative use should consider baseline bleeding risk, organ function, platelet status, disease/treatment factors and exposure time. No validated model available. | Source named major and clinically relevant non-major bleeding and used prophylactically treated patients as denominator. Named standards, post-exposure window, severity/counting rules and adjudication are proposed. | Proposed: capture during care, verify after discharge/death, aggregate monthly. Hospital quality team coordinates extraction/audit; local staff assignment must be agreed. |
| C3.3 — In-hospital VTE-related mortality | Original framework lists hospital-related VTE mortality; no operational definition or formula was provided in thesis Chapter 5. | Proposed: admissions ending in in-hospital death for which objectively confirmed hospital-acquired VTE is adjudicated as the primary or an important contributing cause. Do not equate any death in a patient with VTE with VTE-related death. | Proposed: same admission cohort as C3.1. Exclude deaths attributable only to VTE present on admission and deaths after discharge. Report uncertain attribution separately. | Index admission through in-hospital death or discharge. Count each qualifying admission once. Denominator is the eligible admission cohort, not only patients developing VTE. | Proposed: death/discharge summaries, objective VTE confirmation, clinical timeline and available autopsy findings. Two clinician reviewers independently assess attribution; third adjudicates disagreement. Record certainty/supporting evidence. | VTE must be objectively confirmed and adjudicated as primary or an important contributing cause of in-hospital death. | Lower is desirable, but crude rates should not be used for institutional ranking. Comparative use requires validated adjustment for baseline case mix plus observation time and ascertainment. No method validated in this study. | Entire operational specification, including clarified name, is proposed during revision and was not assessed in the original Delphi consultation. | Proposed: capture during care, verify after discharge/death, aggregate monthly. Hospital quality team coordinates extraction/audit; local staff assignment must be agreed. |
| References supporting proposed refinements — [1] Lyman GH, et al. American Society of Hematology 2021 guidelines for management of venous thromboembolism: prevention and treatment in patients with cancer. Blood Adv. 2021;5:927–974. [2] Schulman S, Kearon C. Definition of major bleeding in clinical investigations of antihemostatic medicinal products in non-surgical patients. J Thromb Haemost. 2005;3:692–694. [3] Kaatz S, Ahmad D, Spyropoulos AC, Schulman S. Definition of clinically relevant non-major bleeding in studies of anticoagulants in atrial fibrillation and venous thromboembolic disease in non-surgical patients. J Thromb Haemost. 2015;13:2119–2126. | | | | | | | | | |
